# Comprehensive Functional Characterization and Clinical Interpretation of 20 Splice-Site Variants of the *RAD51C* Gene

**DOI:** 10.3390/cancers12123771

**Published:** 2020-12-15

**Authors:** Lara Sanoguera-Miralles, Alberto Valenzuela-Palomo, Elena Bueno-Martínez, Patricia Llovet, Beatriz Díez-Gómez, María José Caloca, Pedro Pérez-Segura, Eugenia Fraile-Bethencourt, Marta Colmena, Sara Carvalho, Jamie Allen, Douglas F. Easton, Peter Devilee, Maaike P. G. Vreeswijk, Miguel de la Hoya, Eladio A. Velasco

**Affiliations:** 1 Splicing and Genetic Susceptibility to Cancer, Instituto de Biología y Genética Molecular, Consejo Superior de Investigaciones Científicas (CSIC-UVa), 47003 Valladolid, Spain; lara.sanoguera@uva.es (L.S.-M.); valenzuela.larioja@gmail.com (A.V.-P.); elena.bueno@uva.es (E.B.-M.); bdgomez@ibgm.uva.es (B.D.-G.); frailebe@ohsu.edu (E.F.-B.); 2 Molecular Oncology Laboratory CIBERONC, Hospital Clinico San Carlos, IdISSC (Instituto de Investigación Sanitaria del Hospital Clínico San Carlos), 28040 Madrid, Spain; patriciallovet@gmail.com (P.L.); pedro.perez@salud.madrid.org (P.P.-S.); martacolmena@hotmail.com (M.C.); 3 Instituto de Biología y Genética Molecular, Consejo Superior de Investigaciones Científicas (CSIC-UVa), 47003 Valladolid, Spain; mj.caloca@csic.es; 4 Knight Cancer Research Building, 2720 S Moody Ave, Portland, OR 97201, USA; 5 Centre for Cancer Genetic Epidemiology, Department of Public Health and Primary Care, University of Cambridge, Cambridge CB1 8RN, UK; sc2017@medschl.cam.ac.uk (S.C.); jma73@medschl.cam.ac.uk (J.A.); dfe20@medschl.cam.ac.uk (D.F.E.); 6 Leiden University Medical Center, Department of Human Genetics, 2300RC Leiden, The Netherlands; P.Devilee@lumc.nl (P.D.); M.P.G.Vreeswijk@lumc.nl (M.P.G.V.)

**Keywords:** breast cancer, ovarian cancer, susceptibility genes, *RAD51C*, genetic variants, splicing, aberrant splicing, VUS, functional assay, minigene, clinical interpretation

## Abstract

**Simple Summary:**

Genetic variants in more than 10 genes are known to confer moderate to high risks to breast and/or ovarian cancers (BC/OC). In the framework of the international project BRIDGES, a panel of 34 known or suspected BC/OC genes has been sequenced in 60,466 breast cancer patients and 53,461 controls. In this work, we focus on BRIDGES variants detected in the *RAD51C* gene and their impact on the gene expression step known as splicing (intron removal), whose alteration is a relevant disease mechanism. For this purpose, we bioinformatically analyzed 40 *RAD51C* variants from the intron/exon boundaries, 20 of which were selected. Then, we developed a biotechnological tool, called splicing reporter minigene, containing *RAD51C* exons 2 to 8 where any variant can be introduced by site-directed mutagenesis and functionally assayed in MCF-7 cells under the splicing perspective. Nineteen variants impaired splicing, 18 of which induced severe splicing anomalies. Finally, they were clinically interpreted according to strict guidelines whereby 15 variants were classified as Pathogenic/Likely Pathogenic, so they are clinically actionable. Therefore, carrier patients and families may benefit from tailored prevention protocols and therapies.

**Abstract:**

Hereditary breast and/or ovarian cancer is a highly heterogeneous disease with more than 10 known disease-associated genes. In the framework of the BRIDGES project (Breast Cancer Risk after Diagnostic Gene Sequencing), the *RAD51C* gene has been sequenced in 60,466 breast cancer patients and 53,461 controls. We aimed at functionally characterizing all the identified genetic variants that are predicted to disrupt the splicing process. Forty *RAD51C* variants of the intron-exon boundaries were bioinformatically analyzed, 20 of which were selected for splicing functional assays. To test them, a splicing reporter minigene with exons 2 to 8 was designed and constructed. This minigene generated a full-length transcript of the expected size (1062 nucleotides), sequence, and structure (Vector exon V1- *RAD51C* exons_2-8- Vector exon V2). The 20 candidate variants were genetically engineered into the wild type minigene and functionally assayed in MCF-7 cells. Nineteen variants (95%) impaired splicing, while 18 of them produced severe splicing anomalies. At least 35 transcripts were generated by the mutant minigenes: 16 protein-truncating, 6 in-frame, and 13 minor uncharacterized isoforms. According to ACMG/AMP-based standards, 15 variants could be classified as pathogenic or likely pathogenic variants: c.404G > A, c.405-6T > A, c.571 + 4A > G, c.571 + 5G > A, c.572-1G > T, c.705G > T, c.706-2A > C, c.706-2A > G, c.837 + 2T > C, c.905-3C > G, c.905-2A > C, c.905-2_905-1del, c.965 + 5G > A, c.1026 + 5_1026 + 7del, and c.1026 + 5G > T.

## 1. Introduction

Genetic variants in more than 10 genes are known to confer moderate to high risks to breast and/or ovarian cancers (BC/OC) and explain 5% to 10% of all breast cancers and approximately 20% of all ovarian cancers [1,2]. Most of these genes encode for tumor suppressor proteins that play a role in repair of DNA double-strand (DSB) breaks by homologous recombination (HR). In addition to the main breast cancer genes, *BRCA1* [MIM #113705] [3] and *BRCA2* [MIM #600185] [4], inactivating mutations in *ATM* [MIM #607585], *BARD1* [MIM#601593], *BRIP1* [MIM#605882], *CHEK2* [MIM #604373], *PALB2* [MIM #610355], *RAD51C* [MIM#602774], and *RAD51D* [MIM#602954], among others, confer risk to breast and/or ovarian cancer [1,2,5,6].

Loss-of-function variants in *RAD51C* and *RAD51D* increase the risk of breast and ovarian cancer, but the same has not been demonstrated for other *RAD51* paralogs, or for *RAD51* itself that plays a major role in HR repair [7,8,9,10,11]. Likewise, bi-allelic *RAD51C* (or *FANCO*) deleterious variants have been found in Fanconi Anemia patients [12]. RAD51C participates in the recruitment of RAD51 to DNA damage sites and the stabilization of RAD51 nucleofilaments as part of the BCDX2 complex (RAD51B, RAD51C, RAD51D, and XRCC2). It is also involved in the resolution of Holliday junctions interacting with XRCC3 resulting in the CX3 complex, and recently, it was demonstrated that RAD51C interacts directly with PALB2, a key protein in HR [13,14,15,16,17]. Furthermore, RAD51C has been reported to facilitate ATM-dependent CHEK2 phosphorylation, allowing the activation of CHEK2, another important regulator of the cellular response to DNA damage [18,19].

The detection of germ-line pathogenic variants in these cancer susceptibility genes can contribute to improve the prevention, therapy, and surveillance of breast/ovarian cancer patients, as well as to a better knowledge of BC/OC genetics. Unfortunately, a large fraction of variants is classified as variants of uncertain clinical significance (VUS). Since the association with cancer risk is unknown for these variants, this complicates genetic counseling and the clinical management of patients. Multifactorial likelihood approaches, together with functional studies of variants, can facilitate their interpretation [20,21,22]. Variants of disease-genes are typically assessed according to their predicted impact on protein translation, so protein truncating variants (frameshift and nonsense) are usually classified as damaging variants. However, variants might also have an impact on RNA expression and, e.g., disrupt transcription initiation, miRNA regulation, or splicing [23,24,25,26,27].

Pre-mRNA splicing is an essential gene expression mechanism, whereby introns are excised and exons are consecutively joined to produce the mature mRNA. The splicing motifs include the core consensus sequences (5′ and 3′ splice sites -5′SS and 3′SS-, the polypyrimidine tract, and the branchpoint) and exonic or intronic splicing enhancers and silencers [28]. Variants in these *cis*-motifs may lead to abnormal events such as exon skipping, intron retention, inclusion of pseudoexons, or the use of alternative splice sites [29]. These generate aberrant transcripts which may be associated with a genetic disorder [21,30,31,32]. According to the Human Gene Mutation Database (accessed on 27 November 2019) around 9% (23354/269419) of reported disease-causing mutations impair splicing, although some authors suggested that up to 50% of all human disease mutations impair splicing [33,34].

Given the low precision of in silico analysis tools that predict the impact of candidate variants on RNA splicing, the exact consequences of these genetic changes must be verified in functional assays [35,36]. The most suitable method to determine whether a particular variant affects splicing is the direct analysis of blood RNA from heterozygous carriers (either patients or healthy relatives), although access to blood RNA samples is not always feasible in the diagnostic routine [37,38,39]. Even if available, the assessment of the transcripts derived from the variant allele is hampered by the presence of the wild type one. One possible alternative strategy is to use minigene assays, which have been proven to represent a robust tool for assessing the pathogenicity of potential spliceogenic variants [40,41,42,43].

Multigene panel testing is a cost- and time-effective option to evaluate genes and genetic variants that may be associated with a risk of cancer, and is becoming widely used in clinical practice. Our study was conducted in the context of the BRIDGES project (Breast Cancer After Diagnostic Gene Sequencing; https://bridges-research.eu/) where a panel of 34 known or suspected breast cancer susceptibility genes were sequenced in 60,466 cases and 53,461 controls [44]. Here, we bioinformatically analyze 40 variants from the intron/exon boundaries of the *RAD51C* gene identified in BRIDGES subjects. Twenty variants are selected and functionally tested by minigene assays.

## 2. Results

### 2.1. Bioinformatics Analysis

We identified in BRIDGES patients and controls a total of 40 different variants located at *RAD51C* exon/intron boundaries (see Methods). These variants were bioinformatically analyzed with Max Ent Scan (MES) according to the standards indicated in Materials and Methods. Twenty variants were selected for further analysis, based on their predicted impact on splicing (Table 1 and Table S1). Of the 20 selected variants, eleven variants were predicted to impair the 3′SS, and the other nine were predicted to impair the 5′SS. Six variants (c.405-6T > A, c.571 + 4A > G, c.706-2A > C, c.706-2A > G, c.966-2A > G, and c.966-2A > T) were predicted to impair the SS and simultaneously create a de novo SS. Variants c.146-3C > T, c.1026 + 5_1026 + 7del, and c.1026 + 5G > T did not produce significant MES score changes (≥15%), but they affected the conserved nucleotides of the splice sites. The MES value of the exon 8 donor site (2.0) was below the default threshold (3.0), so NNSplice calculations for variants c.1026 + 5_1026 + 7del and c.1026 + 5G > T were used instead (0.8→ <0.1).

### 2.2. Functional Analysis

**Figure 1:**
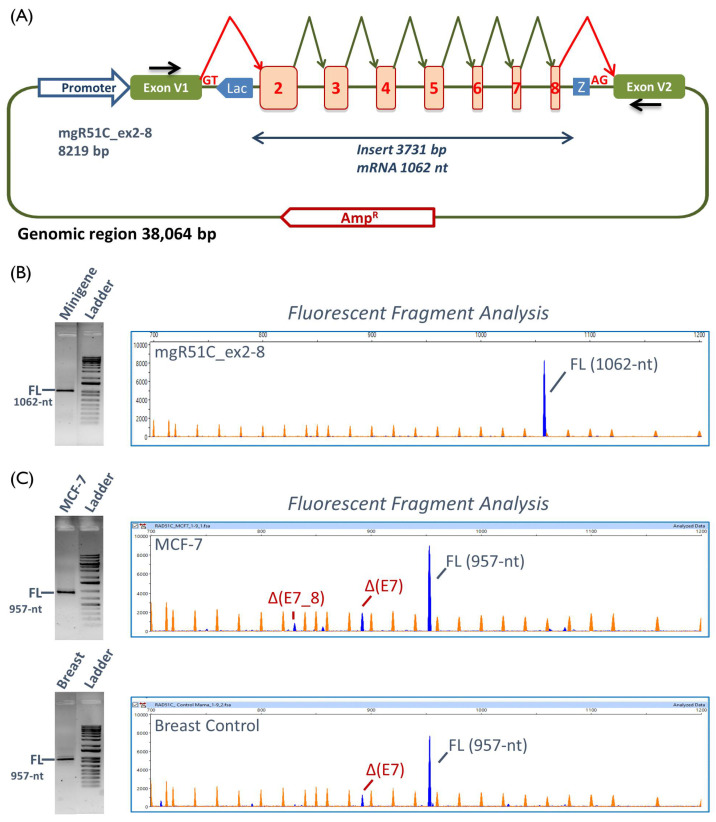
Structure of the minigene mgR51C_ex2-8 and functional validation. (**A**) Schematic representation of the *RAD51C* minigene with exons 2 to 8. Exons are indicated by boxes, broken arrows indicate the expected splicing reactions in eukaryotic cells and black arrows locate specific vector RT-PCR primers. (**B**) Functional assay of the wild type minigene mgR51C_ex2-8. cDNAs were amplified with primers SD6-PSPL3_RTFW and RTpSAD-RV (full-length transcript V1-*RAD51C* ex2-8-V2 = 1062 nt). The RT-PCR product was run by agarose gel electrophoresis (left) and fluorescent capillary electrophoresis (right), where the full-length transcript is shown as a blue peak and the LIZ1200 size standard as orange/faint peaks. (**C**) Agarose gel (left) and fluorescent capillary electrophoresis (right) of transcripts produced by MCF-7 cells (above) and human breast RNA (below). cDNAs were amplified with primers RTR51C_ex1-FW and RTR51C_ex9-RV (full-length transcript = 957nt). FAM-labelled products (blue peaks) were run with LIZ1200 (orange peaks) as the size standard. FL, full-length transcript.

Since all candidate variants were located in exons 2 to 8 (Table 1), we designed a 3731-bp insert containing these seven exons (Figure S1) and cloned this insert into the pSAD vector [41], representing the minigene mgR51C_ex2-8 (Figure 1A). This clone produced a full-length transcript in MCF-7 cells of an expected size (1062 nt), sequence, and structure (V1-*RAD51C*_ex2 to ex8-V2) (Figure 1B), so it was suitable to assess a possible effect of the variants on pre-mRNA splicing. The wild type (wt) construct also generated residual amounts (1.4%) of an unknown 1106-nt transcript that could not be characterized. To identify physiological alternative splicing events, RNA from the host cells (MCF-7) and from the human breast control were analyzed by RT-PCR as well. The expected full-length transcript (957-nt) was detected by fluorescent fragment electrophoresis together with some alternative splicing isoforms, of which exon 7 skipping was the main event (Figure 1C).

**Figure 2:**
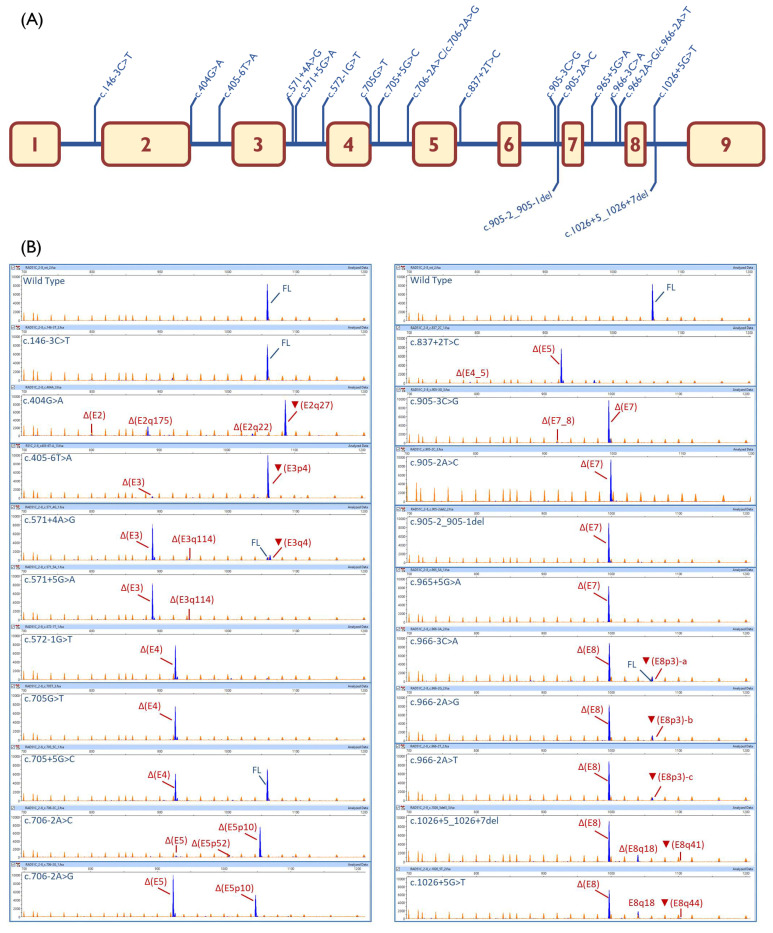
Splicing functional assays of selected *RAD51C* variants. (**A**) Map of tested variants. (**B**) fluorescent fragment analysis of transcripts generated by the wild type and mutant minigenes. cDNAs were amplified with primers SD6-PSPL3_RTFW and RTpSAD-RV (full-length transcript V1-*RAD51C* ex2-8-V2 = 1062 nt). FAM-labelled products (blue peaks) were run with LIZ1200 (orange peaks) as the size standard. For transcript descriptions see Table S2; FL, full-length transcript.

The 20 selected variants were genetically engineered into the wt minigene and then were introduced into MCF-7 cells. Nineteen variants (95%) impaired splicing, 18 of which produced no trace or residual amounts of the full-length transcript (Table 1; Figure 2B). Eight variants affected the classical ±1, 2 positions of the 5′ and 3′SS (c.572-1G > T, c.706-2A > C, c.706-2A > G, c.837 + 2T > C, c.905-2A > C, c.905-2_905-1del, c.966-2A > G, and c.966-2A > T), five changed the +5 position (c.571 + 5G > A, c.705 + 5G > C, c.965 + 5G > A, c.1026 + 5_1026 + 7del, and c.1026 + 5G > T), two modified the last exon nt (c.404G > A and c.705G > T), another two substituted the -3 nt (c.905-3C > G and c.966-3C > A), one disrupted the +4 nt (c.571 + 4A > G), and another one altered the polypyrimidine tract (c.405-6T > A). Only variant c.146-3C > T did not disrupt splicing.

### 2.3. Transcript Analysis

High sensitivity fluorescent fragment analysis allowed us to detect at least 35 transcripts (from 1 to 5 transcripts per variant), 22 of which could be characterized (Table S2, Table 1). Sixteen transcripts introduced premature termination codons (PTC), including 11 predicted to undergo NMD (PTC-NMD transcripts) and 5 disrupting the reading-frame but not predicted to undergo NMD (PTC transcripts). On the other hand, six RNA isoforms kept the open reading-frame, but five of them were minor. Δ(E5) was the most abundant in-frame transcript induced by c.706-2A > G and c.837 + 2T > C (65.4% and 89.3%, respectively). *RAD51C* exon 5 encodes for 44 amino acids, 26 of which are strictly conserved in vertebrates and contain the Walker-B domain between (p.238–242; Figure S2) [45], which plays a relevant role in RAD51C function. Δ(E8q18) was produced by variants c.1026 + 5_1026 + 7del and c.1026 + 5G > T (13.8% and 18.7%, respectively). This transcript encodes for a deletion of six amino acids (Val337 to Lys342, of which only Ile 341 is strictly conserved in vertebrates), which removes six out of the seven amino acids of the essential β-strand-8, suggesting a plausible protein dysfunction (Figure S2). However, no pathogenic missense mutations have been recorded at the ClinVar database in this region, so we cannot confirm that transcript Δ(E8q18) encodes for inactive RAD51C. Of note, variants c.966-3C > A, c.966-2A > G, and c.966-2A > T of the exon 8 3′SS produced three different versions of a 3-nt intronic insertion (acceptor shift; Table 1): ▼(E8p3)-a (r. [966-3c > a,965_966ins966-3_966-1]; 9.7%), ▼(E8p3)-b (r. [966-2a > g,965_966ins966-3_966-1]; 11.0%), and ▼(E8p3)-c (r. [966-2a > u,965_966ins966-3_966-1]; 5.9%), respectively. These would provoke three different effects on protein translation, i.e., p.Arg322dup, p.Arg322delinsSerGly and p.Arg322delinsSerTrp, respectively. Arg322 is strictly conserved, indicating that this residue might be important for protein function (Figure S2). On the other hand, three missense changes have been reported in ClinVar at codon 322 (p.Arg322Lys, p.Arg322Thr, and p.Arg322Ser), all of them classified as VUS, so protein dysfunctionality by any of these three transcripts could not be supported. The remaining in-frame transcript Δ(E3q114) showed a relative proportion below 5% in variants c.571 + 4A > G and c.571 + 5G > A, where 12 out of the 38 deleted amino acids are strictly conserved (Figure S2).

### 2.4. ACMG/AMP-Like Classification of RAD51C Variants Based on PS3/BS3 Functional Evidence

On the basis of the data acquired using minigene analysis, the ACMG/AMP-like classification approach classifies 15 variants as pathogenic/likely pathogenic and 5 variants as of uncertain significance (Table 2; Methods; Figure S3). Incorporating splicing functional data into the ACMG/AMP framework proved to be non-trivial and raised several relevant issues, including the identification of what we think are internal inconsistences of the framework (see Discussion).

## 3. Discussion

Massive parallel sequencing of breast and/or ovarian cancer genes has allowed the genetic testing of thousands of patients in a high throughput and cost-effective strategy. The goal of the BRIDGES initiative was to firmly establish the breast cancer association of genes tested by commercial multigene panels with the narrowest confidence intervals of risk estimates currently available. BRIDGES analyzed 34 known or suspected BC genes that were sequenced in 60,466 patients and 53,461 controls [44]. Nonsense, frameshift, and ±1, 2 splice site variants (sometimes collectively referred to as protein truncating variants or PTVs) are usually assumed to be pathogenic or likely pathogenic. This assumption might work well for certain epidemiological studies but cannot be taken for granted in the clinic (e.g., spliceogenic variants, including ±1, 2 splice site variants, are not necessarily pathogenic, as they may cause in-frame alterations preserving function). Many other variants (e.g., rare missense changes) are considered VUS, due to their unknown impact on gene function and disease risk [48]. In fact, clinical management of VUS carriers (and non-carrier relatives) is complex, since risk evaluation is solely based on family history [49,50].

The *RAD51C* gene was one of the 34 genes analyzed by BRIDGES given its role in breast and ovarian cancer [6,51]. A statistically significant association for PTVs has been found for ER-negative breast cancer and breast and ovarian cancer [44,52]. In this work, we have carried out the most comprehensive splicing study of germline variants of *RAD51C* to date. Forty variants located within the intron/exon boundaries were selected and analyzed by MES or NNSplice. In keeping with the standards indicated in Materials and Methods, 20 candidate variants were chosen (Table 1) for subsequent RNA assays.

In the absence of patient RNA, splicing reporter minigenes provide a straightforward and robust method for the initial characterization of putative spliceogenic variants for several reasons. The assay (i) uses a simple and clean analysis of a single mutant allele; (ii) is performed in a cell type relevant for the disease; (iii) circumvents the NMD interference with the use of an inhibitor; (iv) uses a single construct for testing multiple variants, among other benefits of this technology. Here, we envisioned a construct that contained a synthesized insert with seven (exons 2–8) out of the nine exons of the *RAD51C* gene, so that all the selected variants (Figure 2A) could be evaluated in one single minigene.

Remarkably, all but one variant disrupted splicing, underlining the specificity of our criteria. MES or NNSplice predicted correctly an effect on RNA splicing (either splice-site disruptions or significant score reductions) in 19 variants (Table 1). Only one variant, c.146-3C > T, did not alter splicing, indeed, the MES score was just slightly reduced (−8.5%) because the most frequent −3 nucleotide (C) is substituted by the second most frequent one (T). However, other -3 non-conservative changes in which the nucleotide substitution was different, such as c.905-3C > G and c.966-3C > A, caused total or almost total splicing disruptions. Likewise, a double effect was precisely predicted by MES for c.405-6T > A: 3′SS disruption and generation of a strong de novo 3′SS 4-nt upstream that, in fact, was mainly used by the splicing machinery (▼(E3p4), 95.2%). MES did not identify the exon 8 donor site, although the NNSplice did. In this case, both +5 variants (c.1026 + 5_1026 + 7del and c.1026 + 5G > T) totally disrupted splicing without any trace of the full-length isoform. Conversely, another +5 variant (c.705 + 5G > C) yielded 51.6% of the full-length isoform with a relatively low MES decrease (−20.8%). It is also worthy to mention that c.571 + 4A > G slightly reduced the MES score (−22.5%) but the resultant mutant donor site was still strong (MES = 8.1). However, this change induced an almost complete aberrant splicing with a residual amount of the full-length transcript (5.4%). Finally, the different splicing outcomes of the two changes at the same position, c.706-2A > C and -2A > G, should be highlighted (Table 1). Variant c.706-2A > C mainly caused the use of a cryptic 3′SS 10-nt downstream (Δ(E5p10); 91.4%), while c.706-2A > G mainly generated Δ(E5) (65.4%) but also Δ(E5p10) (33.5%). However, MES scores of the cryptic 3′SS of both changes (3.3 vs. 3.2) were low and not significantly different. One possible explanation could be that the c.706-2A > C is a purine to pyrimidine change that would strengthen the polypyrimidine tract of the internal cryptic acceptor site 10-nt downstream (used in Δ(E5p10)), whereas c.706-2A > G (purine to purine) would not.

Given this and the unpredictability of splicing outcomes, with 35 different transcripts, RNA assays are strongly recommended to investigate the impact of genetic variants on splicing. Fluorescent capillary electrophoresis of the RT-PCR products also offered high resolution and sensitivity, being capable of distinguishing isoforms that differ only in a few nucleotides [53], such as the full-length and ▼(E8p3)-a,b,c transcripts that just contain a 3-nt insertion.

Interestingly, 12 transcripts (▼(E2q27), Δ(E2q175), Δ(E2q22), Δ(E2), Δ(E3), Δ(E4), Δ(E4_5), Δ(E5), Δ(E7), Δ(E7_8), Δ(E8), and▼(E8p3)) had been previously characterized as naturally occurring isoforms of *RAD51C* [54], suggesting that physiological alternative events may somehow predict variant splicing profiles [55,56,57]. Moreover, minigene assays are capable of mimicking pathological patterns of variants. Thus, minigene experiments reproduced previous results of patient RNA assays of several variants inducing very similar or even identical outcomes: c.571 + 4A > G (Δ(E3)) [58], c.706-2A > G (Δ(E5)) [59], c.905-2_905-1del (Δ(E7)) [60], and c.1026 + 5_1026 + 7del (Δ(E8)) [61]. Moreover, variants c.837 + 2T > C and c.905-3C > G/c.905-2A > C mimicked previous results of c.837 + 1G > A and c.905-2A > G of the same splice sites, respectively [62,63]. Finally, variants c.404G > C/G > T, at the same position as c.404G > A, promoted the use of the same cryptic splice site 27-nt downstream (▼(E2q27)) of the canonical donor site [64]. Altogether these results lend support to the reproducibility of the minigene approach. However, while in patient samples, the major and apparently unique aberrant transcript of each of the variants c.571 + 4A > G, c.706-2A > G, and c.1026 + 5_1026 + 7del was the main outcome in minigene assays (Δ(E3)-76.5%, Δ(E5)-65.4%, and Δ(E8)-78.0%, respectively), the minor minigene transcripts were not detected in patient RNAs (Table 1). These slight variations may be due to several reasons, including: (i) tissue-specific alternative splicing, since patient RT-PCRs are usually performed from blood RNA; (ii) the high sensitivity of the fluorescent fragment analysis, which allows the identification of rare isoforms; (iii) the use of NMD inhibitors in minigene experiments (patient samples are not usually NMD-inhibited), which improves the detection of low-abundant PTC-transcripts; (iv) the interference of the wild type allele in patient samples; (v) the high transcription rate triggered by a strong minigene SV40 promoter [38]. Likewise, the wild type construct did not exactly replicate the splicing profile of MCF-7 or control breast samples that showed minor alternative transcripts (Figure 1C). Hence, other factors should be considered, such as the absence of the natural genomic context in the minigene that actually contains shortened introns 2, 3, 4, 5, 6, 7, and 8 (Supplementary Figure S1). Therefore, we might speculate that the absence of putative regulatory intronic elements and the natural exon/intron architecture might somewhat influence splicing outcomes of the wild type and mutant minigenes [65].

### Clinical Interpretation of Variants

The clinical interpretation of variants cannot be done solely on the basis of the functional data presented in this manuscript. From a clinical perspective, the data presented here are to assist in classifying genetic variants. Yet, the analysis of spliceogenic variants is an especially challenging and laborious mission. The presence of numerous *RAD51C* abnormal transcripts and the production of several transcripts by many variants are proofs of this arduous undertaking. From a simple functional viewpoint, the biological indicators of pathogenicity of a particular variant are the strong reduction of the expression of wild type transcript and the presence of severe splicing anomalies that are predicted to result in protein truncation or loss of critical protein domains. On this basis, 18 variants with severe splicing anomalies (Table 1) should be classified as deleterious or likely deleterious.

However, more complex and comprehensive guidelines have been developed for the clinical interpretation of variants, such as those of the ACMG-AMP [66]. Here, we propose a clinical classification of our findings on the basis of these guidelines. Overall, we think that our ACMG/AMP-like classification of 20 *RAD51C* pre-selected variants based on minigene data is rigorous, with most variants placed in the pathogenic/likely pathogenic category, but highlighting as well up to four variants (c.705 + 5G > C, c.966-3C > A, c.966-2A > G, c.966-2A > T) that despite being spliceogenic, require further studies to be definitely classified.

We would like to highlight as well that, at some point, our classification is based on decisions not necessarily shared by other experts in the field (e.g., replacing in silico predictions by functional evidence rather than combining both, see rationale below and in Supplementary Methods). For that reason, others may propose a different clinical classification. In turn, this highlights a relevant issue in variant classification, namely, the lack of standardization.

Accordingly, our minigene-based ACMG/AMP-like classification approach (Table 2) was not intended to produce a definitive (i.e., authoritative) clinical classification of these variants (a prerequisite for that will be the completion of the ClinGen expert panel adaptation of the ACMG/AMP rules to *RAD51C*), but rather to highlight the complexity of determining the appropriate aggregate strength of combining predictive and functional splicing types of evidence into the ACMG/AMP classification framework without introducing inconsistences into the system [67].

Internal inconsistences that we have identified in the ACMG/AMP framework are: (i) GT-AG ± 1, 2 variants producing PTC-NMD transcripts being more easily classified as pathogenic (PVS1 + PS3 = Pathogenic) than nonsense/indels variants introducing equivalent PTC-NMD alterations (PVS1 + ? = Pathogenic), and (ii) GT-AG ±1, 2 variants being more easily classified as pathogenic that other spliceogenic variants producing identical RNA outputs (PVS1+ PS3 = Pathogenic vs. PS3 + PP3 = Uncertain Significance). Further, we think that a system granting likely pathogenic classification for rare GT-AG ± 1, 2 variants (PM2 + PVS1 = likely pathogenic) fails by discouraging RNA analyses.

In the present study, we propose addressing these issues by a somewhat radical approach: replacing in silico predictions by functional evidence (rather than combining both). We think that this approach: (i) avoids the internal inconsistences already mentioned, and (ii) recognizes the fact that predictive and functional splicing pieces of evidence are not truly independent from each other. Implicitly, the ACMG/AMP classification framework assumes that each piece of evidence is independent [68], an assumption hardly met by the predictive and functional criteria as most functional analyses are performed in pre-selected variants on the basis of bioinformatics predictions such as the present study.

The ClinGen *CDH1* expert panel has proposed to use PVS1_Strong (rather than PVS1) for GT-AG ± 1, 2 variants and combine these with RNA (PS3) or association (PS4) data to reach a pathogenic classification [69]. In a second iteration of the rules (www.clinicalgenome.org/affiliation/50014/), the authors refine the approach by stating that for PVS1_Strong variants (GT-AG ± 1, 2), PS3_moderate (rather than PS3) should be applied.

While the suggestion of “downgrading” the loss-of-function prediction for GT-AG ± 1, 2 variants (and encouraging RNA analyses) is appealing to us, the approach does not eliminate internal inconsistences for GT-AG ± 1, 2 vs. other PTC-NMD variants (PVS1_Strong + PS3_moderate = Likely Pathogenic vs. PVS1only = uncertain significance) and does not even address the issue for spliceogenic variants other than GT-AG ± 1, 2. Further, nothing is said about the appropriate strength of combining computational and functional splicing data if the evidence codes go in opposite directions.

In our approach, the computational evidence does not contribute to the final clinical classification of functionally validated spliceogenic variants, but we do acknowledge a fundamental role for these predictions in selecting and prioritizing variants for subsequent splicing analyses. Indeed, we recommend running bioinformatic splicing predictions for all genetic variants regardless of their nature and/or location (i.e., nonsense, in-frame, and frameshift indels and synonymous, non-synonymous, and intronic variants). Further, once a variant is selected for splicing analysis, the predictions have a role in designing and/or validating the corresponding assays. For instance, a negative experimental result (no splicing effect) in a variant with strong computational evidence might points towards a sub-optimal experimental design (e.g., multi-exon skipping is missed due to wrong selection of primers). Further on, a positive result (splicing alteration) for a variant with no strong computational evidence may suggest that it is not the presumed variant under investigation but another variant in *cis* (e.g., a deep intronic variant) that is causing the splicing alteration.

The “quality control” role of computational evidences is probably more relevant for assays performed in RNA from carriers than in minigene-based assays (e.g., in the latter approach there is no doubt about the variant under investigation). Yet, we argue that the concordance with computational evidence (as observed in the present study) is also relevant to consider minigene outputs strong (or very strong) evidence towards pathogenicity.

Ultimately, validation of the pathogenicity will need to be based on the observed risk associated with the variants—either through case-control or family-based studies. It will be extremely challenging to evaluate risk for individual variants, since they are very rare, but it is possible in principle to evaluate the classification system as a whole. Furthermore, in BRIDGES, these spliceogenic variants account for 44.9% of all patients carrying a pathogenic/likely pathogenic variant (data not shown), indicating that a high proportion of *RAD51C* breast cancer risk-associated alleles displays splicing defects, as previously described for *BRCA1* and *BRCA2* [21].

## 4. Materials and Methods

### 4.1. Ethics Approval

Ethical approval for this study was obtained from the Ethics Committee of the Spanish National Research Council-CSIC (28/05/2018).

### 4.2. Variant and Transcript Annotations

BRIDGES sequencing data [44] identified a total of 40 different variants located at *RAD51C* splice sites (SS), defined for the purpose of the present study as: (i) intron/exon (IVS-10_IVS-1/2nt) boundaries (3′SS), and (ii) exon/intron (2nt/IVS + 1_IVS + 10) boundaries (5′SS). Variants and alternative transcripts were annotated according to the Human Genome Variation Society (HGVS) guidelines on the basis of the *RAD51C* GenBank sequence NM_058216.3. To simplify transcript annotation, we identified them with a shortened code that combines the following symbols [56,70]: ∆ (skipping of exonic sequences), ▼ (inclusion of intronic sequences), E (exon), p (acceptor shift), q (donor shift). When necessary, the number of deleted or inserted nucleotides is indicated. For example, ▼ (E2q27) indicates the use of an alternative donor site downstream of exon 2 causing a 27-nt intron insertion.

### 4.3. Bioinformatics Analysis

All 40 *RAD51C* variants from the intron-exon boundaries were analyzed to identify potential splicing variants using splice site prediction software (Table S1). Mutant and wild type sequences were analyzed with the Max Ent Scan (MES) algorithm of Human Splicing Finder 3.1 [71,72], except for exon 8 donor variants that were analyzed by NNSplice [73] because this site was not detected by MES. Potential spliceogenic variants were selected according to the following criteria: (i) splice site disruption at the AG/GT positions; (ii) important MES score changes (≥15%) [35,74]; (iii) creation of de novo splice sites; (iv) regardless of computer predictions, variants at other conserved positions of the acceptor (Y_11_NCAG|G) and donor (MAG|GTRAGT) consensus sequences, such as Pyrimidine to Purine changes or deletions at the polypyrimidine tract, nucleotide substitutions of a conserved nt at the intronic positions −3C, +3R, +4A, +5G, +6T, as well as the first (G) and the last three nucleotides of the exon (M, A, G).

### 4.4. Minigene Construction and Mutagenesis

**Figure 3:**
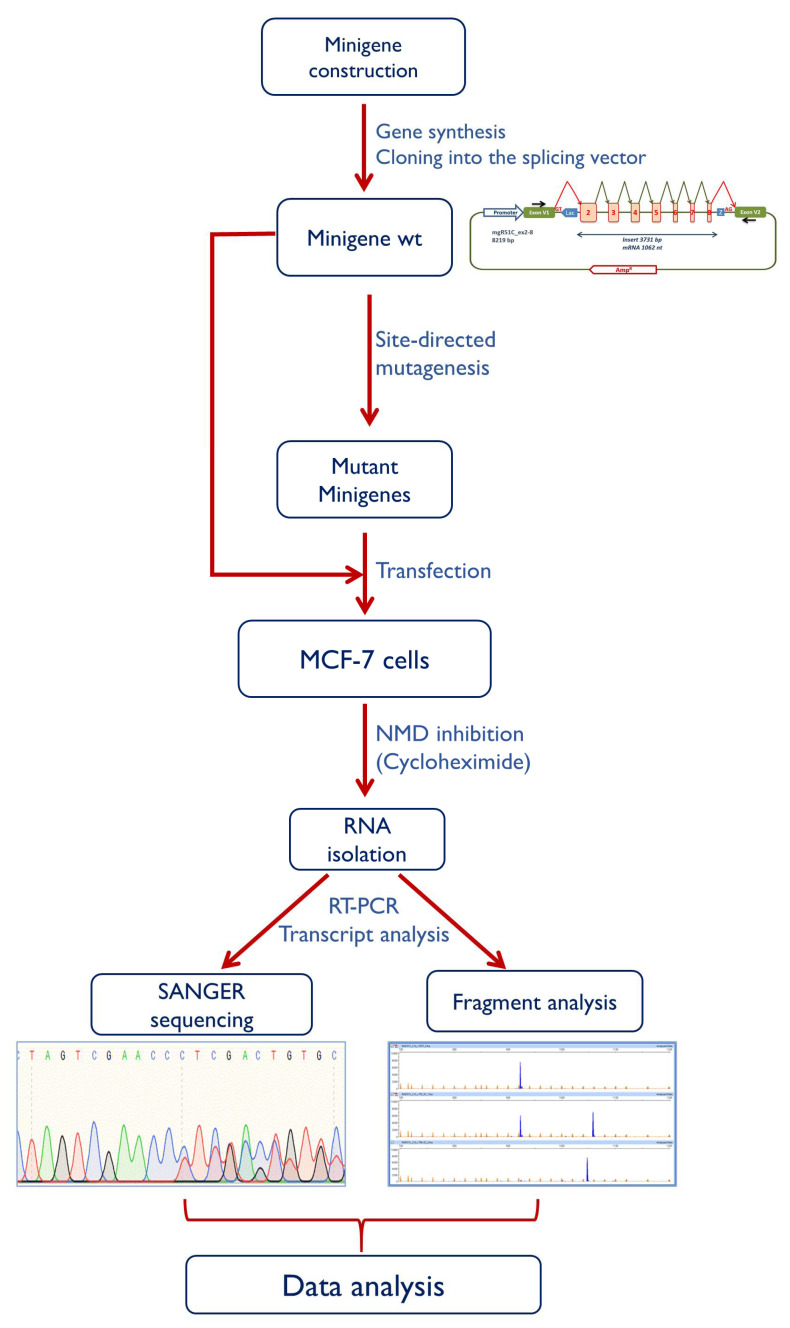
Workflow of the minigene protocol. The basic assay includes the following steps: (1) minigene construction; (2) site-directed mutagenesis; (3) transfection of the wild type and mutant minigenes; (3) inhibition of nonsense-mediated decay and RNA purification; (4) transcript sequencing and fragment analysis by fluorescent capillary electrophoresis; (5) data interpretation.

*RAD51C* has 9 exons but all the potential spliceogenic variants from BRIDGES subjects were located in exons 2 to 8. Therefore, an insert (3731 bp) with exons 2 to 8 and their respective flanking intronic sequences was designed in our laboratory and then synthesized at the Genewiz facility (Genewiz, South Plainfield, NJ, USA) (Figure S1). This fragment was cloned into the splicing vector pSAD (Patent P201231427-CSIC) [41,75] between the restriction sites *Bam*HI and *Eco*RI. The wild type minigene mgR51C_ex2-8 was used as template to generate 20 candidate BRIDGES DNA variants (Table S2) with the QuikChange Lightning kit (Agilent, Santa Clara, CA, USA). All constructs were confirmed by sequencing (Macrogen, Madrid, Spain). The whole protocol is outlined in Figure 3.

### 4.5. Transfection of Eukaryotic Cells

Approximately 2 × 10^5^ MCF-7 cells were grown to 90% confluency in 0.5 mL of medium (MEM, 10% Fetal Bovine Serum, 1% nonessential amino acids, 2mM Glutamine and 1% Penicillin/Streptomycin; Sigma-Aldrich, St. Louis, MO, USA) in 4-well plates (Nunc, Roskilde, Denmark). Cells were transfected with 1 μg of the wt and mutant minigenes using 2 μL of Lipofectamine LTX (Life Technologies, Carlsbad, CA, USA). To inhibit nonsense-mediated decay (NMD), cells were treated with cycloheximide 300 μg/mL (Sigma-Aldrich, St. Louis, MO, USA) for 4 h just before RNA extraction. RNA was purified with the Genematrix Universal RNA Purification Kit (EURx, Gdansk, Poland) including on-column DNase I digestion.

### 4.6. Reverse Transcription Polymerase Chain Reaction and Fragment Analysis

Retrotranscription was carried out with 400 ng of RNA and the RevertAid First Strand cDNA Synthesis Kit (Life Technologies, Carlsbad, CA, USA), using the vector-specific primer RTPSPL3-RV (5′-TGAGGAGTGAATTGGTCGAA-3′). Samples were incubated at 42 °C for 1 h, followed by 5 min at 70 °C. Then, 40 ng of cDNA (final volume of 50 µl) were amplified with SD6-PSPL3_RT-FW (5′-TCACCTGGACAACCTCAAAG-3′) and RTpSAD-RV (Patent P201231427) (size 1062 nt) using Platinum-Taq DNA polymerase (Life Technologies). Samples were denatured at 94 °C for 2 min, followed by 35 cycles of 94 °C/30 sec, 60 °C/30 sec, and 72 °C (1 min/kb), and a final extension step at 72 °C for 5 min. RT-PCR products were sequenced as previously indicated.

In order to quantify all transcripts relatively to each other, semi-quantitative fluorescent RT-PCRs were performed in triplicate with primers PSPL3_RT-FW and RTpSAD-RV (FAM-labelled) and Platinum Taq DNA polymerase (Life Technologies, Carlsbad, CA, USA) under the above standard conditions except that 26 cycles were herein applied [31,41]. FAM-labeled products were run with LIZ-1200 Size Standard at the Macrogen facility and analyzed with the Peak Scanner software V1.0. Only peak heights ≥50 RFU (Relative Fluorescence Units) were considered. Furthermore, MCF-7 and Human Breast Total RNAs (Agilent, cat. no. 540045, discontinued) were retrotranscribed with primer RTR51C_ex9-RV (5′- ACATGCAGAAGTAACAACAG-3′) and then amplified with primers RTR51C_ex1-FW (5′-GAACTCCTAGAGGTGAAAC-3′) and again RTR51C_ex9-RV labelled with FAM (amplicon length: 957 bp) in the same above PCR conditions except that the annealing temperature was set at 58 °C. Mean peak areas of three independent experiments of each variant were used to calculate the relative proportions of each transcript and standard deviations.

### 4.7. ACMG/AMP-Like Classification of 20 RAD51C Variants Based on PS3/BS3 Functional Evidence

Since no ClinGen *RAD51C* Expert panel specifications of the American College of Medical Genetics and Genomics and the Association for Molecular Pathology (ACMG/AMP) variant curation guidelines are currently available (www.clinicalgenome.org/), we performed a tentative classification (ACMG/AMP-like) based on: (i) generic ACMG/AMP guidelines [66]; (ii) specific aspects of the ClinGen Sequence Variant Interpretation Working Group (ClinGen-SVI) recommendations for interpreting the loss-of-function PVS1 and functional PS3/BS3 evidence codes [67,76]; (iii) some non-gene specific approaches developed by the ClinGen *CDH1* variant curation expert panel [69], and (iv) expert judgment.

In addition to PS3/BS3 (functional evidence, in this case based on splicing data obtained from minigene analysis), only the rarity code (PM2) made a major contribution to the classification process. In a subset of variants, association with disease (PS4), and detected in *trans* with a pathogenic variant in Fanconi Anemia patients’ (PM3) codes (see Table 2 and Methods for further details), also contributed. Of note, we excluded the use of predictive evidence codes (i.e., PVS1/PP3) from our classification approach because (i) splicing predictive and functional evidence are not independent from each other, and (ii) incorporating both types of evidence into the framework creates internal inconsistences (see Discussion).

## 5. Conclusions

We have shown that aberrant splicing of *RAD51C* represents a relevant pathogenic mechanism in breast cancer susceptibility. The functional study of variants provides critical data that may increase the number of families that may benefit from preventive or therapeutic measures. In this regard pSAD-derived minigenes have been proven as robust and high capacity approaches for the primary characterization of variant-associated defective splicing, since they replicate splicing results of patient RNA, as we have shown in *RAD51C* and other disease genes [57,77,78,79]. By these means and the application of ACMG-AMP-based criteria, we have classified 15 *RAD51C* variants as pathogenic or likely pathogenic, which constitute the largest number of spliceogenic variants of this gene reported so far.

## Figures and Tables

**Table 1:** Bioinformatics analysis and splicing outcomes of *RAD51C* canonical splice variants.

Variant (HGVS) ^1^	Bioinformatics ^2^	Transcripts			
		Canonical	PTC ^3^	In-Frame	Uncharacterized
Wild type		98.6% ± 0.2%			1106-nt (1.4% ± 0.2%)
c.146-3C > T	[↓]3′SS (9.5→8.7)	100%			
c.404G > A	[−]5′SS (4.8→−3.5)	-	▼(E2q27): 69.3% ± 2.9% Δ(E2q175): 19.9% ± 0.6% Δ(E2q22): 4.3% ± 0.5% Δ(E2): 2.4% ± 0.2%		913-nt (4.1% ± 3.0%)
c.405-6T > A	[−]3′SS (7.7→2.2) [+] 3′SS (8.6) 4-nt upstream	-	▼(E3p4):95.2%± 1.6% Δ(E3): 4.8% ± 1.6%		
c.571 + 4A > G	[↓]5′SS (10.5→8.1) [+] 5′SS (5.5) 4-nt downstream	5.4% ± 0.1%	Δ(E3): 76.5% ± 0.3% ▼(E3q4): 11.6% ± 0.2%	Δ(E3q114): 4.0 ± 0.0%	808-nt (1.4% ± 0.0%) 774-nt (1.1% ± 0.0%)
c.571 + 5G > A	[↓] 5′SS (10.5→5.8)	-	Δ(E3): 91.5% ± 0.3%	Δ(E3q114): 4.8 ± 0.2%	808-nt (1.6% ± 0.0%) 917-nt (1.1% ± 0.1%) 774-nt (1.0% ± 0.0%)
c.572-1G > T	[−]3′SS (7.4→−1.2)	-	Δ(E4): 93.4% ± 0.2%		1005-nt (3.3% ± 0.1%) 1058-nt (3.3% ± 0.1%)
c.705G > T	[−]5′SS (9.1→2.6)	-	Δ(E4): 100%		
c.705 + 5G > C	[↓]5′SS (9.1→7.2)	51.6% ± 2.4%	Δ(E4): 48.4% ± 2.4%		
c.706-2A > C	[−]3′SS (11.1→3.1) [+]3′SS (3.3) 10-nt downstream	-	Δ(E5p10): 91.4% ± 1.5% Δ(E5p52): 1.8% ± 0.9%	Δ(E5): 4.0% ± 0.1%	886-nt (2.8% ± 1.6%)
c.706-2A > G	[−]3′SS (11.1→3.1) [+]3′SS (3.2) 10-nt downstream	-	Δ(E5p10): 33.5% ± 0.2%	Δ(E5): 65.4% ± 0.3%	972-nt (1.1% ± 0.1%)
c.837 + 2T > C	[−]5′SS (8.6→0.8)	-	Δ(E4_5): 2.2% ± 0.1%	Δ(E5): 89.3% ± 0.2%	972-nt (8.5% ± 0.1%)
c.905-3C > G	[−]3′SS (8.2→−4.9)	-	Δ(E7): 98.1% ± 1.0% Δ(E7_8): 1.9% ± 1.0%		
c.905-2A > C	[−]3′SS (8.2→0.1)	-	Δ(E7): 97.4% ± 0.4%		660-nt (2.6% ± 0.4%)
c.905-2_905-1del	[−]3′SS(8.2→0.6)	-	Δ(E7): 100%		
c.965 + 5G > A	[↓]5′SS(8.7→3.8)	-	Δ(E7): 100%		
c.966-3C > A	[−]3′SS (7.3→4.4)	2% ± 1.7%	Δ(E8): 86.8% ± 3.2%	▼(E8p3)-a: 9.7% ± 0.4%	881-nt (1.5% ± 1.4%)
c.966-2A > G	[−]3′SS (7.3→−0.7) [+]3′SS(7) 3-nt upstream	-	Δ(E8): 86.7% ± 0.5%	▼(E8p3)-b:11.0% ± 0.4%	881-nt (1.2% ± 0.0%) 940-nt (1.1% ± 0.2%)
c.966-2A > T	[−]3′SS (7.3→−1.1) [+]3′SS(7.6) 3-nt upstream	-	Δ(E8): 89.1% ± 0.3%	▼(E8p3)-c:5.9% ± 0.1%	881-nt (2.8% ± 0.3%) 940-nt (2.2% ± 0.0%)
c.1026 + 5_1026 + 7del	[−]5′SS(2→?) (NNSplice: 0.8→ <0.1)	-	Δ(E8): 79.5% ± 1.4% ▼(E8q41): 3.3% ± 0.2%	Δ(E8q18):13.8% ± 0.7%	881-nt (2% ± 0.6%) 778-nt (1.4% ± 1.6%)
c.1026 + 5G > T	[−]5′SS(2→?) (NNSplice: 0.8→ <0.1)	-	Δ(E8): 78.0% ± 0.5% ▼(E8q44): 1.4% ± 0.2%	Δ(E8q18):18.7% ± 0.5%	881-nt (1.9% ± 0.2%)

^1^ Variants without any trace (or ≤5%) of the full-length transcript are underlined. ^2^ [−] site disruption; [+] new site; [↓] reduction of MES score. ^3^ PTC: Premature Termination Codon; Δ, loss of exonic sequences; ▼ inclusion of intronic sequences; E (exon), p (acceptor shift), q (donor shift). When necessary, the exact number of nt inserted or deleted is indicated.

**Table 2:** Proposed clinical classification of *RAD51C* variants according to ACMG/AMP-based criteria.

c.HGVS ^1^	Clinvar ^2^	PVS1 ^3^	PP3/BP4 ^4^	PS3/BS3 ^5^	PS4 ^6^	PM2 ^7^	PM ^8^	Proposed pSAD-Based ACMG/AMP-Like Variant Classification ^9^
c.146-3C > T	Conflicting (*) LB (2), VUS, (2)	N/A	(−4%) N/A	BS3	N/A	(4/303,851) N/A	N/A	(BS3 only) Uncertain Significance
c.404G > A	LP (**)	N/A	(−99.5%) PP3	PS3_VS	N/A	(1/300,225) PM2	N/A	(PS3_VS + PM2) Likely Pathogenic
c.405-6T > A	VUS (*)	N/A	(−79%) PP3	PS3_VS	N/A	(0/304,932) PM2	N/A	(PS3_VS + PM2) Likely Pathogenic
c.571 + 4A > G	Conflicting (*) LB (1), LP(1), VUS (6)	N/A	(−30.5%) PP3	(88%VS + 4%S + 5%N/A) PS3	N/A	(1/84,873) PM2	N/A	(PS3 + PM2) Likely Pathogenic ^10^
c.571 + 5G > A	VUS (**)	N/A	(−33.9%) PP3	(95% vs. + 5%S) PS3_VS	PS4	(8/336,321) N/A	PM3 ^11^	(PS3_VS + PS4 + PM3) Pathogenic
c.572-1G > T	not reported	PVS1	N/A	PS3_VS	N/A	(1/304,681) PM2	N/A	(PS3_VS + PM2) Likely Pathogenic
c.705G > T	VUS (**)	N/A	(−75.8%) PP3	PS3_VS	N/A	(2/304,499) PM2	N/A	(PS3_VS + PM2) Likely Pathogenic ^10^
c.705 + 5G > C	not reported	N/A	(−16.8%) PP3	(48%VS + 52% N/A) N/A	N/A	(1/304,406) PM2	N/A	(PM2 only) Uncertain Significance
c.706-2A > C	LP (**)	PVS1	N/A	(95%VS + 5% S) PS3_VS	N/A	(0/336,207) PM2	N/A	(PS3_VS + PM2) Likely Pathogenic
c.706-2A > G	P/LP (**)	PVS1	N/A	(34%VS + 65% S) PS3	PS4 ^12^	(10/336,207) N/A	N/A	(PS3 + PS4) Pathogenic
c.837 + 2T > C	LP (**)	PVS1	N/A	(90% S + 2% VS) PS3	N/A	(0/304,832) PM2	N/A	(PS3 + PM2) Likely Pathogenic
c.905-3C > G	not reported	N/A	(−92.8%) PP3	PS3	N/A	(1/336,187) PM2	N/A	(PS3 + PM2) Likely Pathogenic
c.905-2A > C	P/LP (**)	PVS1	N/A	PS3	PS4	(5/336,191) N/A	N/A	(PS3 + PS4) Pathogenic
c.905-2_905-1del	P/LP (**)	PVS1	N/A	PS3	PS4	(4/304,579) N/A	N/A	(PS3 + PS4) Pathogenic
c.965 + 5G > A	LP(1); VUS(2)	N/A	(−59.9%) PP3	PS3	N/A	(2/304,579) PM2	N/A	(PS3 + PM2) Likely Pathogenic
c.966-3C > A	not reported	N/A	(−35.3%) PP3	(90%S + 10%N/A) N/A	N/A	(1/304,818) PM2	N/A	(PM2 only) Uncertain Significance
c.966-2A > G	LP (*)	PVS1	N/A	(90%S + 10%N/A) N/A	N/A	(0/304,818) PM2	N/A	(PM2 only) Uncertain Significance
c.966-2A > T	not reported	PVS1	N/A	(90%S + 10%N/A) N/A	N/A	(0/304,818) PM2	N/A	(PM2 only) Uncertain Significance
c.1026 + 5_1026 + 7del	P/LP (**)	N/A	(−98.8%) PP3	PS3	PS4	(6/304,853) N/A	N/A	(PS3 + PS4) Pathogenic
c.1026 + 5G > T	not reported	N/A	(−98.8%) PP3	PS3	N/A	(0/304,840) PM2	N/A	(PS3 + PM2) Likely Pathogenic

^1^ NM_058216.3. ^2^ ClinVar as 10/07/2020. LB (likely benign), VUS (variant uncertain significance), LP (Likely Pathogenic), P (Pathogenic). ClinVar review status is summarized as follows; two stars (**) for criteria provided + multiple submitters + no conflicts, and one star (*) for criteria provided + single submitter, or for conflicting interpretations of pathogenicity. In the latter case, the number of submitters supporting each interpretation is indicated. ^3^ PVS1 (pathogenic very strong), ^4^ PP3/BP4 (computational evidence supports a deleterious effect/suggest no impact). ^5^ PS3/PS3_VS/BS3 (functional data supports damaging effect/very strongly supports damaging effect/shows no effect). pSAD read-outs (transcripts) were interpreted as per ClinGen-SVI PVS1 recommendations. If transcripts with different evidence strengths were observed, the approximated % is shown. In these cases, final PS3 strength was based on expert judgment (see methods). ^6^ PS4 (strong pathogenic based on association studies). For association evidence, we compared MAF in BC cases (60,466 BRIDGES BC cases) and controls (53,461 BRIDGES controls + gnomADv2.1 NFE). ^7^ PM2 (moderate pathogenic based on rarity). For rarity evidence, we counted alleles in 53,461 BRIDGES controls + gnomADv2.1 global. ^8^ PM3 (moderate pathogenic based on detection in trans with pathogenic variant in a recessive disorder) ^9^ Predictive evidence codes (PVS1/PP3/BP4) are excluded from our pSAD-based ACMG/AMP-like classification approach (see Discussion). ^10^ ACMG/AMP guidelines are not intended to identify “intermediate risk variants”. Yet, we think that is worth considering this possibility for variants expressing the variable proportion of (likely) functional mRNAs (see Supplementary Methods) ^11^ [46]. ^12^ [47].

## Supplementary Materials

The following are available online at https://www.mdpi.com/2072-6694/12/12/3771/s1, Figure S1. Insert sequence of minigene mgR51C_ex2-8. Figure S2. Amino acid conservation of the deleted in-frame sequences of the anomalous *RAD51C* transcripts Δ(E3q114), ∆(E5) and Δ(E8q18). Figure S3. Loss-of-function annotation of 22 *RAD51C* altered transcripts. Table S1. Bioinformatics analysis of *RAD51C* variants with Max Ent Score. Table S2. Enigma and HGVS annotations according to transcript ENST00000337432.9 of *RAD51C*. Table S3. Mutagenesis primers for *RAD51C* variants. Supplementary Methods. ACMG/AMP-like classification of 20 *RAD51C* variants based on PS3/BS3 functional evidence.

## References

[1] Nielsen F.C., van Overeem Hansen T., Sørensen C.S. (2016). Hereditary breast and ovarian cancer: New genes in confined pathways. Nat. Rev. Cancer.

[2] Schubert S., Luttikhuizen J.L., Auber B., Schmidt G., Hofmann W., Penkert J., Davenport C.F., Hille-Betz U., Wendeburg L., Bublitz J. (2019). The identification of pathogenic variants in BRCA1/2 negative, high risk, hereditary breast and/or ovarian cancer patients: High frequency of FANCM pathogenic variants. Int. J. Cancer.

[3] Miki Y., Swensen J., Shattuck-Eidens D., Futreal P.A., Harshman K., Tavtigian S., Liu Q., Cochran C., Bennett L.M., Ding W. (1994). A strong candidate for the breast and ovarian cancer susceptibility gene BRCA1. Science.

[4] Wooster R., Bignell G., Lancaster J., Swift S., Seal S., Mangion J., Collins N., Gregory S., Gumbs C., Micklem G. (1995). Identification of the breast cancer susceptibility gene BRCA2. Nature.

[5] Buys S.S., Sandbach J.F., Gammon A., Patel G., Kidd J., Brown K.L., Sharma L., Saam J., Lancaster J., Daly M.B. (2017). A study of over 35,000 women with breast cancer tested with a 25-gene panel of hereditary cancer genes. Cancer.

[6] Castéra L., Harter V., Muller E., Krieger S., Goardon N., Ricou A., Rousselin A., Paimparay G., Legros A., Bruet O. (2018). Landscape of pathogenic variations in a panel of 34 genes and cancer risk estimation from 5131 HBOC families. Genet. Med..

[7] Taylor M.R.G., Špírek M., Chaurasiya K.R., Ward J.D., Carzaniga R., Yu X., Egelman E.H., Collinson L.M., Rueda D., Krejci L. (2015). Rad51 Paralogs Remodel Pre-synaptic Rad51 Filaments to Stimulate Homologous Recombination. Cell.

[8] Sullivan M.R., Bernstein K.A. (2018). RAD-ical New Insights into RAD51 Regulation. Genes.

[9] Le Calvez-Kelm F., Oliver J., Damiola F., Forey N., Robinot N., Durand G., Voegele C., Vallée M.P., Byrnes G., Breast Cancer Family Registry (2012). RAD51 and Breast Cancer Susceptibility: No Evidence for Rare Variant Association in the Breast Cancer Family Registry Study. PLoS ONE.

[10] Yang X., Song H., Leslie G., Engel C., Hahnen E., Auber B., Horváth J., Kast K., Niederacher D., Turnbull C. (2020). Ovarian and breast cancer risks associated with pathogenic variants in RAD51C and RAD51D. J. Natl. Cancer Inst..

[11] Lhotova K., Stolarova L., Zemankova P., Vocka M., Janatova M., Borecka M., Cerna M., Jelinkova S., Kral J., Volkova Z. (2020). Multigene panel germline testing of 1333 Czech patients with ovarian cancer. Cancers.

[12] Vaz F., Hanenberg H., Schuster B., Barker K., Wiek C., Erven V., Neveling K., Endt D., Kesterton I., Autore F. (2010). Mutation of the RAD51C gene in a Fanconi anemia-like disorder. Nat. Genet..

[13] Yokoyama H., Sarai N., Kagawa W., Enomoto R., Shibata T., Kurumizaka H., Yokoyama S. (2004). Preferential binding to branched DNA strands and strand-annealing activity of the human Rad51B, Rad51C, Rad51D and Xrcc2 protein complex. Nucleic Acids Res..

[14] Liu Y., Masson J.Y., Shah R., O’Regan P., West S.C. (2004). RAD51C Is Required for Holliday Junction Processing in Mammalian Cells. Science.

[15] Somyajit K., Subramanya S., Nagaraju G. (2010). RAD51C: A novel cancer susceptibility gene is linked to Fanconi anemia and breast cancer. Carcinogenesis.

[16] Suwaki N., Klare K., Tarsounas M. (2011). RAD51 paralogs: Roles in DNA damage signalling, recombinational repair and tumorigenesis. Semin. Cell Dev. Biol..

[17] Park J.Y., Zhang F., Andreassen P.R. (2014). PALB2: The hub of a network of tumor suppressors involved in DNA damage responses. Biochim. Biophys. Acta Rev. Cancer.

[18] Badie S., Liao C., Thanasoula M., Barber P., Hill M.A., Tarsounas M. (2009). RAD51C facilitates checkpoint signaling by promoting CHK2 phosphorylation. J. Cell Biol..

[19] Zannini L., Delia D., Buscemi G. (2014). CHK2 kinase in the DNA damage response and beyond. J. Mol. Cell Biol..

[20] Goldgar D.E., Easton D.F., Deffenbaugh A.M., Monteiro A.N.A., Tavtigian S.V., Couch F.J., Information C., Bic C. (2004). Integrated evaluation of DNA sequence variants of unknown clinical significance: Application to BRCA1 and BRCA2. Am. J. Hum. Genet..

[21] Sanz D.J., Acedo A., Infante M., Durán M., Pérez-Cabornero L., Esteban-Cardeñosa E., Lastra E., Pagani F., Miner C., Velasco E.A. (2010). A high proportion of DNA variants of BRCA1 and BRCA2 is associated with aberrant splicing in breast/ovarian cancer patients. Clin. Cancer Res..

[22] Farrugia D.J., Agarwal M.K., Pankratz V.S., Deffenbaugh A.M., Pruss D., Frye C., Wadum L., Johnson K., Mentlick J., Tavtigian S.V. (2008). Functional Assays for Classification of BRCA2 Variants of Uncertain Significance. Cancer Res..

[23] Fraile-Bethencourt E., Valenzuela-Palomo A., Díez-Gómez B., Infante M., Durán M., Marcos G., Lastra E., Gómez-Barrero S., Velasco E.A. (2018). Genetic dissection of the BRCA2 promoter and transcriptional impact of DNA variants. Breast Cancer Res. Treat..

[24] Brewster B.L., Rossiello F., French J.D., Edwards S.L., Wong M., Wronski A., Whiley P., Waddell N., Chen X., Bove B. (2012). Identification of fifteen novel germline variants in the BRCA1 3’UTR reveals a variant in a breast cancer case that introduces a functional miR-103 target site. Hum. Mutat..

[25] Diederichs S., Bartsch L., Berkmann J.C., Fröse K., Heitmann J., Hoppe C., Iggena D., Jazmati D., Karschnia P., Linsenmeier M. (2016). The dark matter of the cancer genome: Aberrations in regulatory elements, untranslated regions, splice sites, non-coding RNA and synonymous mutations. EMBO Mol. Med..

[26] Burke L.J., Sevcik J., Gambino G., Tudini E., Mucaki E.J., Shirley B.C., Whiley P., Parsons M.T., De Leeneer K., Gutiérrez-Enríquez S. (2018). BRCA1 and BRCA2 5′ noncoding region variants identified in breast cancer patients alter promoter activity and protein binding. Hum. Mutat..

[27] Gelli E., Colombo M., Pinto A., De Vecchi G., Foglia C., Amitrano S., Morbidoni V., Imperatore V., Manoukian S., Baldassarri M. (2019). Usefulness and Limitations of Comprehensive Characterization of mRNA Splicing Profiles in the Definition of the Clinical Relevance of BRCA1/2 Variants of Uncertain Significance. Cancers.

[28] Yoshida K., Ogawa S. (2014). Splicing factor mutations and cancer. Wiley Interdiscip. Rev. RNA.

[29] Canson D., Glubb D., Spurdle A.B. (2020). Variant effect on splicing regulatory elements, branchpoint usage, and pseudoexonization: Strategies to enhance bioinformatic prediction using hereditary cancer genes as exemplars. Hum. Mutat..

[30] Scotti M.M., Swanson M.S. (2016). RNA mis-splicing in disease. Nat. Rev. Genet..

[31] Fraile-Bethencourt E., Díez-Gómez B., Velásquez-Zapata V., Acedo A., Sanz D.J., Velasco E.A. (2017). Functional classification of DNA variants by hybrid minigenes: Identification of 30 spliceogenic variants of BRCA2 exons 17 and 18. PLoS Genet..

[32] Obeng E.A., Stewart C., Abdel-Wahab O. (2019). Altered RNA processing in cancer pathogenesis and therapy. Cancer Discov..

[33] Baralle D., Buratti E. (2017). RNA splicing in human disease and in the clinic. Clin. Sci..

[34] Matlin A.J., Clark F., Smith C.W.J. (2005). Understanding alternative splicing: Towards a cellular code. Nat. Rev. Mol. Cell Biol..

[35] Moles-Fernández A., Duran-Lozano L., Montalban G., Bonache S., López-Perolio I., Menéndez M., Santamariña M., Behar R., Blanco A., Carrasco E. (2018). Computational Tools for Splicing Defect Prediction in Breast/Ovarian Cancer Genes: How Efficient Are They at Predicting RNA Alterations?. Front. Genet..

[36] Tosi M., Stamm S., Baralle D. (2010). RNA splicing meets genetic testing: Detection and interpretation of splicing defects in genetic diseases. Eur. J. Hum. Genet..

[37] Whiley P.J., De La Hoya M., Thomassen M., Becker A., Brandão R., Pedersen I.S., Montagna M., Menéndez M., Quiles F., Gutiérrez-Enríquez S. (2014). Comparison of mRNA splicing assay protocols across multiple laboratories: Recommendations for best practice in standardized clinical testing. Clin. Chem..

[38] Baralle D., Lucassen A., Buratti E. (2009). Missed threads. The impact of pre-mRNA splicing defects on clinical practice. EMBO Rep..

[39] Gaildrat P., Killian A., Martins A., Tournier I., Frébourg T., Tosi M. (2010). Use of splicing reporter minigene assay to evaluate the effect on splicing of unclassified genetic variants. Methods Mol. Biol..

[40] Cooper T.A. (2005). Use of minigene systems to dissect alternative splicing elements. Methods.

[41] Acedo A., Hernández-Moro C., Curiel-García Á., Díez-Gómez B., Velasco E.A. (2015). Functional classification of BRCA2 DNA variants by splicing assays in a large minigene with 9 exons. Hum. Mutat..

[42] Fraile-Bethencourt E., Valenzuela-Palomo A., Díez-Gómez B., Acedo A., Velasco E.A. (2018). Identification of Eight Spliceogenic Variants in BRCA2 Exon 16 by Minigene Assays. Front. Genet..

[43] Baralle D., Baralle M. (2005). Splicing in action: Assessing disease causing sequence changes. J. Med. Genet..

[44] Dorling L., Carvalho S., Allen J., González-Neira A., Luccarini C., Wahlström C., Pooley K.A., Parsons M.T., Fortuno C., Wang Q. (2020). Breast cancer risk genes: Association analysis in more than 113,000 women. N. Engl. J. Med..

[45] Miller K.A., Sawicka D., Barsky D., Albala J.S. (2004). Domain mapping of the Rad51 paralog protein complexes. Nucleic Acids Res..

[46] Jacquinet A., Brown L., Sawkins J., Liu P., Pugash D., Van Allen M.I., Patel M.S. (2018). Expanding the FANCO/RAD51C associated phenotype: Cleft lip and palate and lobar holoprosencephaly, two rare findings in Fanconi anemia. Eur. J. Med. Genet..

[47] Suszynska M., Ratajska M., Kozlowski P. (2020). BRIP1, RAD51C, and RAD51D mutations are associated with high susceptibility to ovarian cancer: Mutation prevalence and precise risk estimates based on a pooled analysis of ~30,000 cases. J. Ovarian Res..

[48] van Marcke C., Collard A., Vikkula M., Duhoux F.P. (2018). Prevalence of pathogenic variants and variants of unknown significance in patients at high risk of breast cancer: A systematic review and meta-analysis of gene-panel data. Crit. Rev. Oncol. Hematol..

[49] Radice P., De Summa S., Caleca L., Tommasi S. (2011). Unclassified variants in BRCA genes: Guidelines for interpretation. Ann. Oncol..

[50] Eccles D.M., Mitchell G., Monteiro A.N.A., Schmutzler R., Couch F.J., Spurdle A.B., Gómez-García E.B., ENIGMA Clinical Working Group (2015). *BRCA1* and *BRCA2* genetic testing—pitfalls and recommendations for managing variants of uncertain clinical significance. Ann. Oncol..

[51] Meindl A., Hellebrand H., Wiek C., Erven V., Wappenschmidt B., Niederacher D., Freund M., Lichtner P., Hartmann L., Schaal H. (2010). Germline mutations in breast and ovarian cancer pedigrees establish RAD51C as a human cancer susceptibility gene. Nat. Genet..

[52] Song H., Dicks E., Ramus S.J., Tyrer J.P., Intermaggio M.P., Hayward J., Edlund C.K., Conti D., Harrington P., Fraser L. (2015). Contribution of Germline Mutations in the RAD51B, RAD51C, and RAD51D Genes to Ovarian Cancer in the Population. J. Clin. Oncol..

[53] Acedo A., Sanz D.J., Durán M., Infante M., Pérez-Cabornero L., Miner C., Velasco E.A. (2012). Comprehensive splicing functional analysis of DNA variants of the BRCA2 gene by hybrid minigenes. Breast Cancer Res..

[54] Brandão R.D., Mensaert K., López-Perolio I., Tserpelis D., Xenakis M., Lattimore V., Walker L.C., Kvist A., Vega A., Gutiérrez-Enríquez S. (2019). Targeted RNA-seq successfully identifies normal and pathogenic splicing events in breast/ovarian cancer susceptibility and Lynch syndrome genes. Int. J. Cancer.

[55] Fackenthal J.D., Yoshimatsu T., Zhang B., de Garibay G.R., Colombo M., De Vecchi G., Ayoub S.C., Lal K., Olopade O.I., Vega A. (2016). Naturally occurring BRCA2 alternative mRNA splicing events in clinically relevant samples. J. Med. Genet..

[56] Lopez-Perolio I., Leman R., Behar R., Lattimore V., Pearson J.F., Castéra L., Martins A., Vaur D., Goardon N., Davy G. (2019). Alternative splicing and ACMG-AMP-2015-based classification of PALB2 genetic variants: An ENIGMA report. J. Med. Genet..

[57] Fraile-Bethencourt E., Valenzuela-Palomo A., Díez-Gómez B., Goina E., Acedo A., Buratti E., Velasco E.A. (2019). Mis-splicing in breast cancer: Identification of pathogenic BRCA2 variants by systematic minigene assays. J. Pathol..

[58] Shirts B.H., Casadei S., Jacobson A.L., Lee M.K., Gulsuner S., Bennett R.L., Miller M., Hall S.A., Hampel H., Hisama F.M. (2016). Improving performance of multigene panels for genomic analysis of cancer predisposition. Genet. Med..

[59] Walsh T., Casadei S., Lee M.K., Pennil C.C., Nord A.S., Thornton A.M., Roeb W., Agnew K.J., Stray S.M., Wickramanayake A. (2011). Mutations in 12 genes for inherited ovarian, fallopian tube, and peritoneal carcinoma identified by massively parallel sequencing. Proc. Natl. Acad. Sci. USA.

[60] Lhota F., Zemankova P., Kleiblova P., Soukupova J., Vocka M., Stranecky V., Janatova M., Hartmannova H., Hodanova K., Kmoch S. (2016). Hereditary truncating mutations of DNA repair and other genes in *BRCA1*/*BRCA2*/*PALB2* -negatively tested breast cancer patients. Clin. Genet..

[61] Golmard L., Caux-Moncoutier V., Davy G., Al Ageeli E., Poirot B., Tirapo C., Michaux D., Barbaroux C., D’Enghien C.D., Nicolas A. (2013). Germline mutation in the RAD51B gene confers predisposition to breast cancer. BMC Cancer.

[62] Coulet F., Fajac A., Colas C., Eyries M., Dion-Minière A., Rouzier R., Uzan S., Lefranc J.-P., Carbonnel M., Cornelis F. (2013). Germline *RAD51C* mutations in ovarian cancer susceptibility. Clin. Genet..

[63] Pelttari L.M., Heikkinen T., Thompson D., Kallioniemi A., Schleutker J., Holli K., Blomqvist C., Aittomäki K., Bützow R., Nevanlinna H. (2011). RAD51C is a susceptibility gene for ovarian cancer. Hum. Mol. Genet..

[64] Neidhardt G., Becker A., Hauke J., Horváth J., Bogdanova Markov N., Heilmann-Heimbach S., Hellebrand H., Thiele H., Altmüller J., Nürnberg P. (2017). The RAD51C exonic splice-site mutations c.404G>C and c.404G>T are associated with familial breast and ovarian cancer. Eur. J. Cancer Prev..

[65] Hertel K.J. (2008). Combinatorial control of exon recognition. J. Biol. Chem..

[66] Richards S., Aziz N., Bale S., Bick D., Das S., Gastier-Foster J., Grody W.W., Hegde M., Lyon E., Spector E. (2015). Standards and guidelines for the interpretation of sequence variants: A joint consensus recommendation of the American College of Medical Genetics and Genomics and the Association for Molecular Pathology. Genet. Med..

[67] Brnich S.E., Abou Tayoun A.N., Couch F.J., Cutting G.R., Greenblatt M.S., Heinen C.D., Kanavy D.M., Luo X., McNulty S.M., Starita L.M. (2020). Recommendations for application of the functional evidence PS3/BS3 criterion using the ACMG/AMP sequence variant interpretation framework. Genome Med..

[68] Tavtigian S.V., Greenblatt M.S., Harrison S.M., Nussbaum R.L., Prabhu S.A., Boucher K.M., Biesecker L.G., ClinGen Sequence Variant Interpretation Working Group (ClinGen SVI) (2018). Modeling the ACMG/AMP variant classification guidelines as a Bayesian classification framework. Genet. Med..

[69] Lee K., Krempely K., Roberts M.E., Anderson M.J., Carneiro F., Chao E., Dixon K., Figueiredo J., Ghosh R., Huntsman D. (2018). Specifications of the ACMG/AMP variant curation guidelines for the analysis of germline CDH1 sequence variants. Hum. Mutat..

[70] Fraile-Bethencourt E., Valenzuela-Palomo A., Díez-Gómez B., Caloca M.J., Gómez-Barrero S., Velasco E.A. (2019). Minigene Splicing Assays Identify 12 Spliceogenic Variants of BRCA2 Exons 14 and 15. Front. Genet..

[71] Desmet F.O., Hamroun D., Lalande M., Collod-Bëroud G., Claustres M., Béroud C. (2009). Human Splicing Finder: An online bioinformatics tool to predict splicing signals. Nucleic Acids Res..

[72] Yeo G., Burge C.B. (2004). Maximum entropy modeling of short sequence motifs with applications to RNA splicing signals. J. Comput. Biol..

[73] Reese M.G., Eeckman F.H., Kulp D., Haussler D. (1997). Improved splice site detection in Genie. J. Comput. Biol..

[74] Houdayer C., Caux-Moncoutier V., Krieger S., Barrois M., Bonnet F., Bourdon V., Bronner M., Buisson M., Coulet F., Gaildrat P. (2012). Guidelines for splicing analysis in molecular diagnosis derived from a set of 327 combined in silico/in vitro studies on BRCA1 and BRCA2 variants. Hum. Mutat..

[75] de Garibay G.R., Acedo A., García-Casado Z., Gutiérrez-Enríquez S., Tosar A., Romero A., Garre P., Llort G., Thomassen M., Díez O. (2014). Capillary electrophoresis analysis of conventional splicing assays: IARC analytical and clinical classification of 31 BRCA2 genetic variants. Hum. Mutat..

[76] Abou Tayoun A.N., Pesaran T., DiStefano M.T., Oza A., Rehm H.L., Biesecker L.G., Harrison S.M., ClinGen Sequence Variant Interpretation Working Group (ClinGen SVI) (2018). Recommendations for interpreting the loss of function PVS1 ACMG/AMP variant criterion. Hum. Mutat..

[77] Lara B., Martínez M.T., Blanco I., Hernández-Moro C., Velasco E.A., Ferrarotti I., Rodriguez-Frias F., Perez L., Vazquez I., Alonso J. (2014). Severe alpha-1 antitrypsin deficiency in composite heterozygotes inheriting a new splicing mutation QOMadrid. Respir. Res..

[78] Gailite L., Valenzuela-Palomo A., Sanoguera-Miralles L., Rots D., Kreile M., Velasco E.A. (2020). UGT1A1 Variants c.864+5G>T and c.996+2_996+5del of a Crigler-Najjar Patient Induce Aberrant Splicing in Minigene Assays. Front. Genet..

[79] Villate O., Ibarluzea N., Fraile-Bethencourt E., Valenzuela A., Velasco E.A., Grozeva D., Raymond F.L., Botella M.P., Tejada M.-I. (2018). Functional Analyses of a Novel Splice Variant in the CHD7 Gene, Found by Next Generation Sequencing, Confirm Its Pathogenicity in a Spanish Patient and Diagnose Him with CHARGE Syndrome. Front. Genet..

