# Supplementary material for: Comprehensive Functional Characterization and Clinical Interpretation of 20 Splice-Site Variants of the RAD51C Gene
Source: Cancers (Basel). 2020 Dec 15;12(12):3771. doi: 10.3390/cancers12123771 (PMC7765170; doi:10.3390/cancers12123771)
Supplement: Supplementary file 1 [file cancers-12-03771-s001.zip › cancers-988386 SM proofreading/cancers-988386 Supplementary_Figures_S1_S2_S3.pdf]

*Bam*HI

GGATCCtcccaaagtactgggattttaggcatgagccactgtgtccggccaaactgaaaaattaaatggttgatagaatgtt
gcattttatgtttctccactcctagcatcactgttgtctacaaattaataaagacaatcgattatcatgttacacttttaa
atctctaaaattagggttctttttttcttattttactttcag**AAGTTGGGATATCTAAAGCAGAAGCCTTAGAAACTCTGCA**
AATTATCAGAAGAGAATGTCTCACAAATAAACCAAGATATGCTGGTACATCTGAGTCACACAAGAAGTGTACAGCACTGGAA **Ex2**
CTTCTTGAGCAGGAGCATACCCAGGGCTTCATAATCACCTTCTGTTCAGCACTAGATGATATTCTTGGGGGTGGAGTGCCCT
TAATGAAAACAACAGAAATTTGTGGTGCACCAGGTGTTGGAAAAACACAATTATGgtaaaataaagtgttctccttttaagg
gtgggtttaataacatattatgaaagtagtattttgtactatcgtcaggaaaccaaataagatatatatgtgctcttaattt
taagtgtgtatgtgcattaaacaaaaattagcttactatttgtgttacttcattatcccttcttgataaatgtatgcaatta
ttctgatgtcgaatccagttgatttacatatagtcgattggttcacattatatctggagtcatgaagagttagacatttctg
ttgccttggggagtatatttacatttataaaactttagtgatacctaacttgtcattatctggagttcaaaaacactacctt
agatcatcatcatgatttggttgtttgtcatctttctgttgacag**TATGCAGTTGGCAGTAGATGTGCAGATACCAGAATGT**
TTTGGAGGAGTGGCAGGTGAAGCAGTTTTTATTGATACAGAGGGAAGTTTTATGGTTGATAGAGTGGTAGACCTTGCTACTG **Ex3**
CCTGCATTCAGCACCTTCAGCTTATAGCAGAAAAACACAAGGGAGAGGgtaagttagtaaatgatcttctttttttctgtat
taataaaagtaatttgcatttgtgcccatctgagacctcagcaacactccatttggaatgtgaagcctaattactggacagt
taactagtgttaaactcttttactactgttacaaaatgagaaatgccacagcctggactggctttttttgttcccctttta
tgtgcacatgtaccctagaactttaattaaaagatcaggaattatgaactgtttatagtaattttgctataatttgtcatct
ttcagttaaagaattttgttatccaaaggagaacattttgttattaaaaagcattgtttttctacaattgccaatacatcca
aacaggtaaaactaattaagagtgttttgttgtttcag**AACACCGAAAAGCTTTGGAGGATTTCACTCTTGATAATATTCTT**
TCTCATATTTATTATTTTCGCTGTCGTGACTACACAGAGTTACTGGCACAAGTTTATCTTCTTCCAGATTTCCTTTCAGAAC **Ex4**
ACTCAAAGgtatgagtcagactactgaaatgtaactaaccaagtatttttgaggtgtttgataagcatgaaaaaataacca
gtacagtagcataaaatcaaagtcaaagccaattgagaaaatctcttccttccttgacctacaatttatttatatttatatt
tgttttgtgtatgtatatgtatatatagtttatgctcagagtaattcatttagcaagtattaattgacacctcctttcctat
atgctatttactgttccaggcattggggatgatatagtaaataagacagaagaatatagtaaataagagagaaggtccctgc
tctcttggagagagagagcatttttattattattattttatttttcgtaacaaatctaatattatctcttctgtatttag**GT**
TCGACTAGTGATAGTGGATGGTATTGCTTTTCCATTTCGTCATGACCTAGATGACCTGTCTCTTCGTACTCGGTTATTAAAT **Ex5**
GGCCTAGCCCAGCAAATGATCAGCCTTGCAAATAATCACAGATTAGCTgtaagtattaactagtgaagagagttttataaca
aagtcaagactgtataaaatgttaatgtctagaaatgtcaaaatagcgtttgcctgacaaataatatagacatgcagttttg
agtaaagttacgtttggttggtttccattaaaaaattctggtcataagtgtcagttttcctggaaaataacaagcataatta
atctgcaacctctgcctgccccaggttcaaatgattctcctgcctcagcctccggagtagctgggattacaggtgcatgcca
ccatgtctggttaattttgtatttttagtagagatggggtttcacaatcttggccagactggtctacttgataattttcaa
agagactcacctaatttcttacattttgtttttgtag**GTAATTTTAACCAATCAGATGACAACAAAGATTGATAGAAATCA** **Ex6**
GGCCTTGCTTGTTCCTGCATTAGgtgggtaattaatcagataaacattttagtttatcacagtttttcttatctctttcatt
tgattctcattgagtactatacgcttcatgaaagcagactgtatttgtcttgttcactggttaatcttagcactaagcacag
taccgttagatactgattgttccttttagttatttaacatttattgagtggccagacactaatgatttgcagtatttcccta
gataggtaaaaaccttgtaatctcaggatattactcattttccaggtttttttgaaagcaagtatactttcgttatgttaaat
taataaagtaagattatatttgatcagaggcgttctgagaaatgtataaccaagtcagtaaggccatatacagttattatgt
tttttactctcag**GGGAAAGTTGGGGACATGCTGCTACAATACGGCTAATCTTTCATTGGGACCGAAAGCAAAG**gtcagtac **Ex7**
agaaacaagttaataactccgaatattgggttaattatactgaatgaacacttacaggtttcttagagctagtcctgtggat
gagatatacagtgacccatgaagtgacactttgttgccttgtctgatatcacctagagcaaacatagaaatgtgttgttat
tactgcatatttagctacataggaaatttttcatataaactctgttatatacatacgggtaatttgaagggtgtattttttaa
tatttctctcctttttgtgttcttagagaaaaaatagaattattaatataataaacctatacatttaaataatgagtttggt
catctgaacttttaattaattaagttcatgtgtttgtatgtatttattctttttctttaagcag**GTTGGCAACATTGTACAA**
GTCACCCAGCCAGAAGGAATGCACAGTACTGTTTCAAATCAAAgtcagtattatttgattagagtgggattttgatattgat **Ex8**
gggcggtaattatctaaagagagaatttacaacttgcttctgtcaacttctgtaggcagcaagcatatttgaggacagcttt
tgatgcttagaacattgtccccaaaatagtagtatatacagaatattgtagtcacaaatggactgaaatttattcttaagag
cagaggaaatacttattttggggtagtgtgttccatgccatatgtaaGAATTC

*Eco*RI

Figure S1. Insert sequence of minigene mgR51C_ex2-8. Exons 2 to 8 are indicated in upper case and cloning sites (*Bam*HI / *Eco*RI) are underlined. Structure of the insert (3,731 bp): *Bam*HI - ivs1 (200 bp) – ex2 (259 bp) – ivs2-1 (200bp) // ivs2-2 (200 bp) – ex3 (167 bp) – ivs3-1 (200 bp) // ivs3-2 (200 bp) – ex4 (134 bp) – ivs4-1 (200 bp) // ivs4-2 (200 bp) – ex5 (132 bp) – ivs 5-1 (200 bp) // ivs5-2 (200 bp) – ex6 (67 bp) – ivs6-1 (200 bp) // ivs6-2 (200 bp) – ex7 (61 bp) – ivs7-1 (200 bp) // ivs7-2 (200 bp) – ex8 (61 bp) – ivs8 (250 bp) – *Eco*RI (shortened introns are indicated by a double slash //)

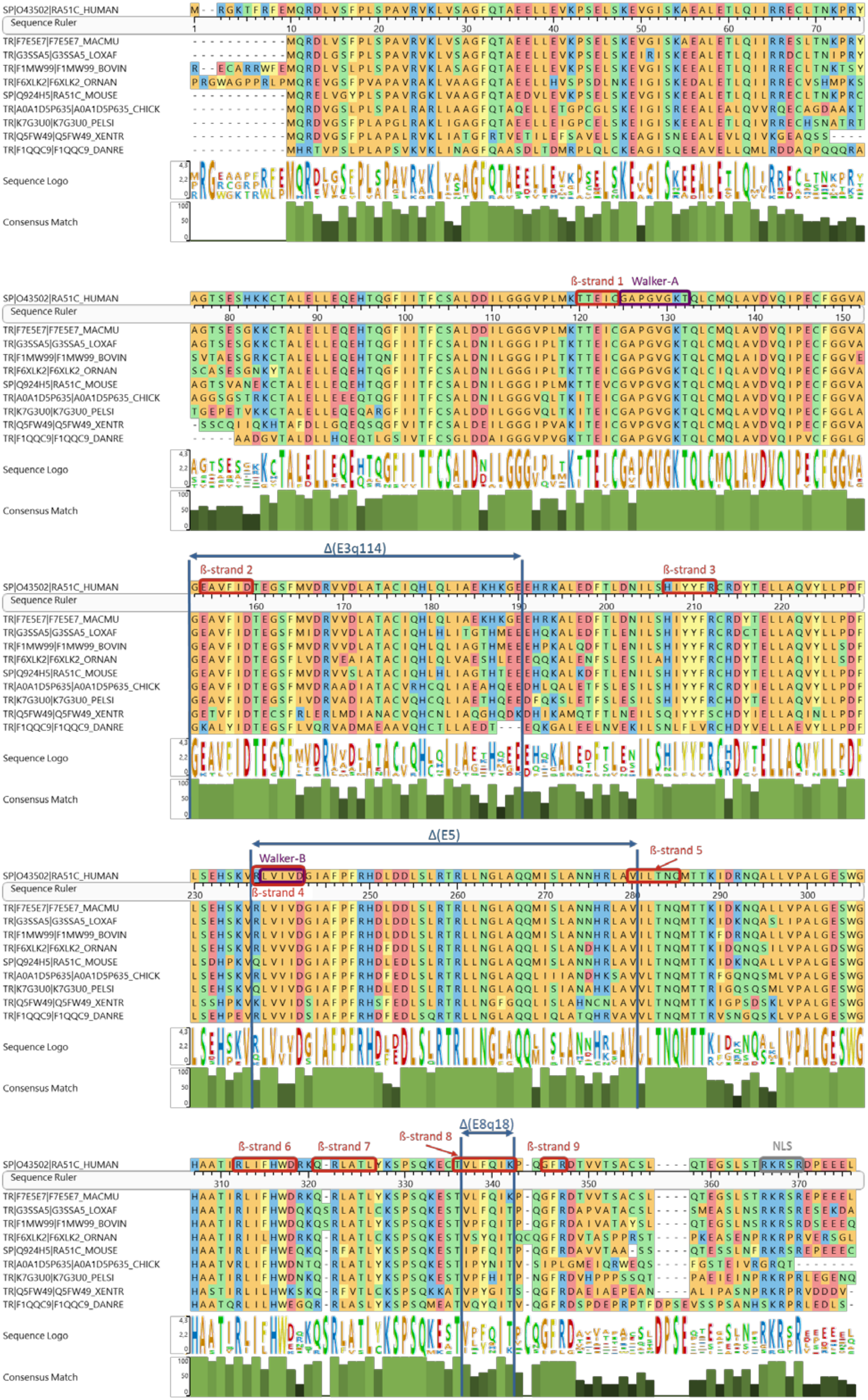

Supplementary Figure S2 (see legend on next page)

Figure S2. **Amino acid conservation of the deleted in-frame sequences of the anomalous *RAD51C* transcripts Δ(E3q114), ∆(E5) and Δ(E8q18).** The deleted in-frame regions are marked in blue stripes and arrows and the key functional domains of RAD51C are denoted as follows: the Walker domains for ATP-hydrolysis are shown in purple boxes, the β-strands are shown in red boxes and the nuclear localization signal (NLS) is displayed in a gray box. Protein sequences were aligned with the Align tool of the Uniprot database (https://www.uniprot.org/align/). The alignment file was visualized with MegAlign Pro version 15.0.0 of DNASTAR's Lasergene software. The conserved residues are highlighted (nonpolar amino acids in orange, polar amino acids in green, polar basic amino acids in blue and polar acidic amino acids in red) and the color intensity of green bars above the protein sequence indicates the degree of conservation of each amino acid. Organisms: Human (*Homo sapiens*); Rhesus Macaque (*Macaca mulatta*: MACMU); African elephant (*Loxodonta africana*: LOXAF); Bovine (*Bos taurus*: BOVIN); Duckbill platypus (*Ornithorhynchus anatinus*: ORNAN); Mouse (*Mus musculus*: MOUSE); Chicken (*Gallus gallus*: CHICK); Chinese Softshell Turtle (*Pelodiscus sinensis*: PELSI); African Clawed Frog (*Xenopus tropicalis*: XENTR); Zebrafish (*Danio rerio*: DANRE).

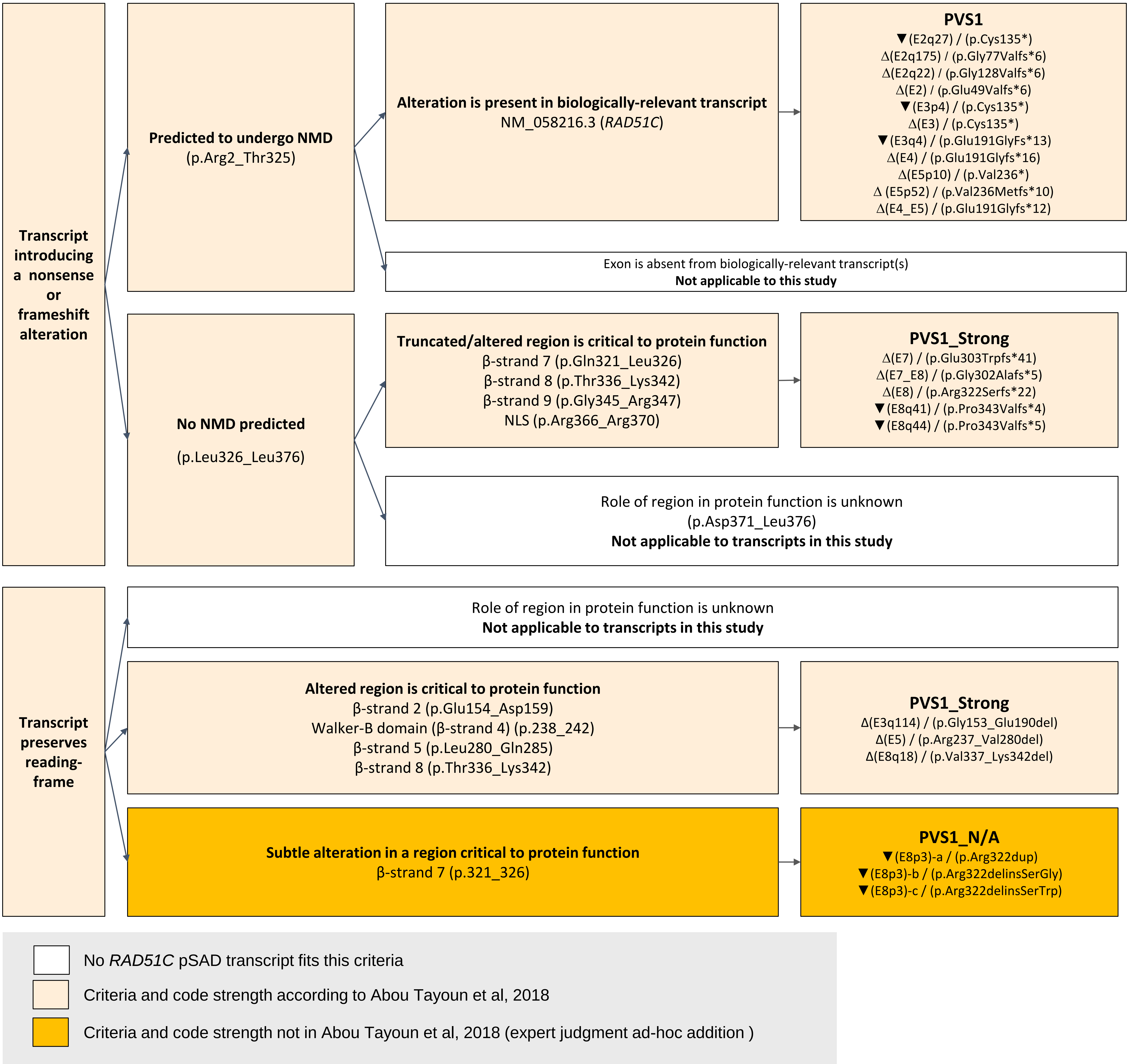

Figure S3. Loss-of-function annotation of 22 *RAD51C* altered transcripts. We have adapted the PVS1 decision tree proposed by Tayoun and col to the specific purpose of determining the strength of the loss-of-function evidence for all 22 *RAD51C* altered transcripts produced by our pSAD analysis. The flowchart indicates the different possibilities, as proposed by Abou Tayoun and col, with the following modification: even if targeting a critical protein region (Miller et al, 2004), we propose being very conservative (PVS1_N/A) for subtle alterations that modify only one or two amino acids.

References
Abou Tayoun, A.N.; Pesaran, T.; DiStefano, M.T.; Oza, A.; Rehm, H.L.; Biesecker, L.G.; Harrison, S.M.; ClinGen Sequence Variant Interpretation Working Group (ClinGen SVI) Recommendations for interpreting the loss of function PVS1 ACMG/AMP variant criterion. Hum. Mutat. 2018, 39, 1517–1524.
Miller, K.A.; Sawicka, D.; Barsky, D.; Albala, J.S. Domain mapping of the Rad51 paralog protein complexes. Nucleic Acids Res. 2004, 32, 169–78.
