# Supplementary material for: Comprehensive Functional Characterization and Clinical Interpretation of 20 Splice-Site Variants of the RAD51C Gene
Source: Cancers (Basel). 2020 Dec 15;12(12):3771. doi: 10.3390/cancers12123771 (PMC7765170; doi:10.3390/cancers12123771)
Supplement: Supplementary file 1 [file cancers-12-03771-s001.zip › cancers-988386 SM proofreading/cancers-988386 Supplementary_Methods - ACMG_AMP interpretation_20 variants-R2.pdf]

SUPPLEMENTAL METHODS.

ACMG/AMP-like classification of 20 *RAD51C* variants based on PS3/BS3 functional evidence.

Predictive splicing codes PVS1/PP3/BP4: Depending on factors such as naturally occurring alternative splicing, exon size, location of cryptic sites and/or the precise location of protein functional domains, not all GT-AG±1,2 variants in *RAD51C* necessarily qualify for PVS1 [1]. Yet, adapting the PVS1 decision tree to the specific case of *RAD51*C is out of the scope of this study (and not relevant to our effort, since we propose excluding the predictive evidence from the classification process once RNA data is available).

The PP3 evidence was added to all variants demonstrating at least a 10% average score (MaxEnt + NNSplice + SSF) decrease as automatically calculated by Alamut Visual version 2.11 (Interactive Biosoftware, Rouen, France). The only exception was PVS1 variants, for which PP3 evidence was not considered to avoid double counting [2]. For variant c.146-3C>T demonstrating an average score decrease of -4%, neither PP3 nor BP4 were considered.

Functional Evidence code PS3/BS3. Several ClinGen-SVI and ClinGen expert panel documents acknowledge the use of minigenes as a source of data for the PS3/BS3 code [2–4]. For the purpose of this study, we consider the pSAD minigene system a well-established functional assay based on: (i) previous data produced by our laboratory with the pSAD system [5,6], (ii) high concordance with previous RT-PCR experiments performed in RNA from carriers of *RAD51C* variants, and (iii) agreement with *in silico* predictions (MES+NNSplice+SSF).

Originally developed by the ClinGen-SVI to decide PVS1 strength for GT-AG±1,2 and other presumed *loss-of-function* variants (Tayoun et al, 2018), we have used the same rationale (see Supplemental Figure 3) to classify the evidence in favor of pathogenicity (very strong, strong, moderate, or none) provided by each individual transcript produced by our pSAD output (minor uncharacterized transcripts representing 1-3% of the overall expression have been excluded from the analysis, see Table 1). Overall, 22 different transcripts were detected, including 11 disrupting reading-frame and predicted to undergo NMD (PTC-NMD transcripts), 5 disrupting reading-frame but not predicted to undergo NMD (PTC transcripts), and 6 preserving reading-frame (in-frame transcripts).

All PTC-NMD transcripts [▼(E2q27) (p.Cys135*), Δ(E2) (p.Glu49Valfs*6), Δ(E2q175) (p.Gly77Valfs*6), Δ(E2q22) (p. Gly128Valfs*6), ▼(E3p4) (p.Cys135*), Δ(E3) (p. Cys135*), ▼(E3q4) (p.Glu191GlyFs*13), Δ(E4) (p. Glu191Glyfs*16), Δ(E4_E5) (p. Glu191Glyfs*12), Δ(E5p10) (p. Val236*), and Δ (E5p52) (p. Val236Metfs*10)] were classified as very strong (loss-of-function) evidence in favor of pathogenicity.

In order to classify PTC and in-frame transcripts, we first defined the following regions critical to RAD51C protein function [7]: (i) Walker-A (p.125-132) and Walker-B domains (p.238-242) essential for ATP-hydrolysis, (ii) ß-strand 1(p.120-124), ß-strand 2 (p.154-159), ß-strand 3 (p.207-212), ß-strand 4 (p.237-242), ß-strand 5 (p.280-285), ß-strand 6 (p.312-318), ß-strand 7 (p.321-326), ß-strand 8 (p.336-342) and ß-strand 9 (p.345-347), as RAD51C proteins lacking any of these nine β-strands are predicted to fail forming the internal β-sheet and collapse, and (iii) the nuclear localization signal (p.366_370, manual assertion based on sequence analysis, UniProtKB-O43502).

Three PTC transcripts [Δ(E7), Δ(E7_E8), and Δ(E8)] were considered to provide strong functional evidence in favor of pathogenicity. The predicted protein products

p.(Glu303Trpfs*41), p.(Gly302Alafs*5), and p.(Arg322Serfs*22), lack β-strands 6 to 9 (7 to 9 in the latter case) and the nuclear localization signal. An identical rationale supported strong evidence in favor of pathogenicity for ▼(E8q41) and ▼(E8q44) PTC transcripts.

Three in-frame transcripts [Δ(E3q114), Δ(E5), and Δ(E8q18)] were considered to provide strong evidence in favor of pathogenicity. The predicted protein products p.(Gly153_Glu190del), p.(Arg237_Val280del), and p.(Val337_Lys342del) lack respectively B-strand 2, Walker-B domain (β-strand 4) plus β-strand 5, and β-strand 8.

Other in-frame transcripts (▼(E8p3) transcripts) were NOT considered to provide any evidence in favor of pathogenicity. The predicted protein product p.Arg322dup in c.966-3C>A carriers, p.(Arg322delinsSerGly) in c.966-2A>G carriers, and p.(Arg322delinsSerTrp) in c.966-2A>T carriers, target the β-strand 7, but deducing a functional impact for such subtle protein changes is not obvious (see Supplemental Figure 3).

For variants producing only one transcript (or different transcripts supporting pathogenicity of equal strength), PS3 annotation of the pSAD output was straightforward. However, for variants producing two or more transcripts providing different levels of evidence, the proper PS3 annotation strength of the pSAD output was not obvious. In these cases, we have applied expert judgment as indicated below (we are not aware of ClinGen-SVI specific recommendations on this topic).

After pSAD analysis, only one *RAD51C* variant (**c.146-3C>T**) demonstrated no splicing alterations (Table 1). Since this is an intronic variant and no functional effect other than a splicing alteration is expected, the negative result was considered a genuine **BS3** functional evidence (this is in full agreement with *CDH1* and *PTEN* expert panel specifications).

Four *RAD51C* variants (**c.404G>A, c.405-6T>A, c.572-1G>T, c.705G>T**) produced only PTC-NMD transcripts (Table 1). Accordingly, the pSAD functional evidence was considered very strong (**PS3_VS**).

Four *RAD51C* variants (**c.905-3C>G, c.905-2A>C, c.905-2_905-1del, and c.965+5G>A**) produced only PTC transcripts (Table 1) supporting strong evidence in favor of pathogenicity. Accordingly, the pSAD functional evidence for these variants was considered strong (**PS3**). Similarly, two variants (**c.1026+5_1026+7del and c.1026+5G>T**) produce only in-frame transcripts supporting strong evidence in favor of pathogenicity (**PS3**).

Two variants (**c.571+5G>A and c.706-2A>C**) produced mostly PTC-NMD transcripts, but also a small proportion (<5%) of in-frame transcripts (Δ(E3q114) and Δ(E5), respectively) (Table 1) that were considered a strong piece of evidence in favor of pathogenicity (Supplemental Figure S3). We propose that the combined functional effect of both transcripts (**95% PS3_VS + 5% PS3**) is best described as very strong (**PS3_VS**).

RAD51C variants **c.571+4A>G**, **c.705+5G>C, c.966-3C>A, c.966-2A>G and c.966-2A>T** were more challenging to interpret. In addition to frankly altered transcripts, they produce variable but non-negligible proportions of canonical and/or near-canonical transcripts (Table 1). In brief: RAD51C c.705+5G>C produced similar amounts of Δ(E4) (PTC-NMD) and canonical transcripts (Table 1); RAD51C c.966-3C>A, c.966-2A>G and c.966-2A>T produced mostly Δ(E8) transcripts, but also 6-11% of ▼(E8p3) transcripts (c.966-3C>A produced in addition ≈2% of canonical transcripts); and RAD51C c.571+4A>G produced mostly Δ(E3) and ▼(E3q4) transcripts, but also ≈4% of Δ(E3q114) and ≈5% of canonical transcripts.

Transcripts ▼(E3p4), (E3), and Δ(E4) provide very strong evidence in favor of pathogenicity while Δ(E3q114) and Δ(E8) provide strong evidence in favor of pathogenicity. Canonical and ▼(E8p3) transcripts do NOT provide any (Supp Table S3). Based on that, we can summarize the pSAD functional evidence for these five variants as follows: (≈48% PS3_VS + ≈52% PS3_N/A) for RAD51C c.705+5G>C; (≈90% PS3 + ≈10% PS3_N/A) for RAD51C c.966-3C>A, c.966-2A>G and c.966-2A>T; and (≈88% PS3_VS + ≈4% PS3 + ≈5% PS3_N/A) for RAD51C c.571+4A>G.

The appropriate PS3 strength of these pSAD functional evidence is far from obvious. Ultimately, the appropriate strength will depend on various aspects of RAD51C biology that, as far as we know, are currently unknown, such as the minimum level of RAD51C activity triggering tumor suppression. Arguable, in individuals expressing higher amount of gene transcripts, even a relatively little fraction of functional mRNAs will be sufficient to trigger tumor suppression activity, further complicating variant classification. At this point in time, we think that the only clinically meaningful approach is to set a very conservative (albeit arbitrary) threshold for potential functional mRNAs expression providing tumor suppressor haplo-sufficiency. In the present study, we have set this arbitrary threshold at 10%. Based on this, we conclude that the appropriate PS3 code strengths are:

PS3_N/A for RAD51C c.705+5G>C (based on up to 52% PS3_N/A), PS3_N/A for RAD51C c.966-3C>A, c.966-2A>G and c.966-2A>T (based on up to10% PS3_N/A), and PS3 for c.571+4A>G (based on ≈5% PS3_N/A, below the threshold, plus ≈88% PS3_VS + ≈4% PS3).

While out of the scope of this study (and not addressed by the current ACMG/AMP guidelines), we think is worth considering the possibility that RAD51C

spliceogenic variants expressing variable proportions of likely functional mRNAs (≈5%-50% in the above examples) are indeed "intermediate risk variants" associated with different risk levels.

Two *RAD51C* variants (**c.706-2A>G and c.837+2T>C**) produce mostly in-frame Δ(E5) transcripts, but also significant [≈33% of Δ(E5p10)] or very low amounts [≈2% of Δ(E4_5)] of PTC-NMD transcripts. In both cases, the appropriate strength of these pSAD functional proofs (summarized as ≈ 65% PS3 + ≈34% PS3_VS and ≈90% PS3 + ≈2% PS3_VS, respectively) is, in our opinion, **PS3**.

Association evidence PS4. We have tested association with breast cancer by comparing allele frequencies in BC carriers (BRIDGES dataset) vs. BRIDGES controls plus gnomAD (NFE sub-population). Statistical analyses were performed using MedCalc online Odds ratio calculator (MedCalc Software, Ostend, Belgium). Statistically significant evidence of association with BC were obtained for c.571+5G>A, (OR=15.1, 95% CI 1.76-129.3, p=0.01), c.905-2A>C (OR=18.1, 95% CI 2.18-150.5, p=0.007), c.905-2_905-1del (OR= 6.9, 95% CI 1.34-35.61, p=0.02), and c.1026+5_1026+7del (OR= 5.1, 95% CI 1.9 to 13.7, p=0.001) (See Table 2 for further details). The *RAD51C* variant c.706-2A>G (that did not reach statistically significant association with BC in our data set) has been demonstrated to associate with ovarian cancer (OR= 10.33; 3.82–27.95; <0.0001) in a recent study [8].

Rarity Evidence PM2. The original ACMG/AMP guidelines defined this rarity code as absent from controls (ExAC, ESP, and/or 1000 genomes project). However, the availability of even larger control datasets (e.g. gnomAD) challenges the view that pathogenic alleles cannot be found in these datasets. Here we follow the rule proposed by the ClinGen CDH1 variant curation expert panel [4] of ≤1 in 100.000 alleles. For that

allele counting, we have combined gnomADv2 and BRIDGES controls cohorts. For variants with 0 alleles in gnomADv2, the number of tested alleles has been deduced from the closest variant reported (in all cases, ≤3-nt apart). Due to poor quality calling in gnomADv2 (only 31412 alleles passed filters), for variant c.571+4A>G we did count only 1 in 84,873 alleles. Yet, based on expert judgment we have considered that this variant qualifies for the rarity code PM2.

In trans with a pathogenic variant in a recessive disorder PM3. The *RAD51C* variant c.571+5G>A had been identified in trans with pathogenic variant c.935G>A (p.Arg312Gln) in a FA patient [9], thus providing a PM3 evidence in favor of pathogenicity.

Frequency greater than expected for disorder BS1. We have calculated the maximum credible variant frequency [10] for a *RAD51C* variant using the online application at http://cardiodb.org/allelefrequencyapp/. We have performed calculations with the following conservative parameters: (i) autosomal dominant, prevalence (breast cancer) at 1:10, allelic heterogeneity at 0.1 (the variant account for 10% of all the pathogenic *RAD51C* alleles), genetic heterogeneity at 0.02 (2% of all breast cancer caused by a *RAD51C* germ-line mutation), and penetrance at 0.2 (20% penetrance based on [11] to provide a Maximum credible population AF: 5e-04 (a maximum of 74 alleles in gnomAD), and (ii) dominant, prevalence (ovarian cancer) at 1:100 (https://seer.cancer.gov/statfacts/html/ovary.html), allelic heterogeneity at 0.1 (the variant account for 10% of all the pathogenic *RAD51C* alleles), genetic heterogeneity at 0.1 (10% of all breast cancer caused by a *RAD51C* germ-line mutation), and penetrance at 0.1 (10% penetrance based on [11]) to provide a Maximum credible population AF: 5e-04 (a maximum of 74 alleles in gnomAD). This analysis shows that the benign code BS1 is not applicable to any of our 20 variants of interest (Table 2 for further details).

Bibliography

1. Abou Tayoun, A.N.; Pesaran, T.; DiStefano, M.T.; Oza, A.; Rehm, H.L.; Biesecker, L.G.; Harrison, S.M.; ClinGen Sequence Variant Interpretation Working Group (ClinGen SVI) Recommendations for interpreting the loss of function PVS1 ACMG/AMP variant criterion. *Hum. Mutat.* **2018**, *39*, 1517–1524.

2. Richards, S.; Aziz, N.; Bale, S.; Bick, D.; Das, S.; Gastier-Foster, J.; Grody, W.W.; Hegde, M.; Lyon, E.; Spector, E.; et al. Standards and guidelines for the interpretation of sequence variants: a joint consensus recommendation of the American College of Medical Genetics and Genomics and the Association for Molecular Pathology. *Genet. Med.* **2015**, *17*, 405–24.

3. Brnich, S.E.; Abou Tayoun, A.N.; Couch, F.J.; Cutting, G.R.; Greenblatt, M.S.; Heinen, C.D.; Kanavy, D.M.; Luo, X.; Mcnulty, S.M.; Starita, L.M.; et al. Recommendations for application of the functional evidence PS3/BS3 criterion using the ACMG/AMP sequence variant interpretation framework On behalf of the Clinical Genome Resource Sequence Variant Interpretation Working Group. **2020**, 1–12.

4. Lee, K.; Krempely, K.; Roberts, M.E.; Anderson, M.J.; Carneiro, F.; Chao, E.; Dixon, K.; Figueiredo, J.; Ghosh, R.; Huntsman, D.; et al. Specifications of the ACMG/AMP variant curation guidelines for the analysis of germline CDH1 sequence variants. *Hum. Mutat.* **2018**, *39*, 1553–1568.

5. Acedo, A.; Hernández-Moro, C.; Curiel-García, Á.; Díez-Gómez, B.; Velasco, E.A. Functional classification of BRCA2 DNA variants by splicing assays in a large minigene with 9 exons. *Hum. Mutat.* **2015**, *36*, 210–21.

6. Fraile-Bethencourt, E.; Díez-Gómez, B.; Velásquez-Zapata, V.; Acedo, A.; Sanz, D.J.; Velasco, E.A. Functional classification of DNA variants by hybrid minigenes: Identification of 30 spliceogenic variants of BRCA2 exons 17 and 18. *PLoS Genet.* **2017**, *13*, e1006691.

7. Miller, K.A.; Sawicka, D.; Barsky, D.; Albala, J.S. Domain mapping of the Rad51 paralog protein complexes. *Nucleic Acids Res.* **2004**, *32*, 169–78.

8. Suszynska, M.; Ratajska, M.; Kozlowski, P. BRIP1, RAD51C, and RAD51D mutations are associated with high susceptibility to ovarian cancer: Mutation prevalence and precise risk estimates based on a pooled analysis of ~30,000 cases. *J. Ovarian Res.* **2020**, *13*.

9. Jacquinet, A.; Brown, L.; Sawkins, J.; Liu, P.; Pugash, D.; Van Allen, M.I.; Patel, M.S. Expanding the FANCO/RAD51C associated phenotype: Cleft lip and palate and lobar holoprosencephaly, two rare findings in Fanconi anemia. *Eur. J. Med. Genet.* **2018**, *61*, 257–261.

10. Whiffin, N.; Minikel, E.; Walsh, R.; O'Donnell-Luria, A.H.; Karczewski, K.; Ing, A.Y.; Barton, P.J.R.; Funke, B.; Cook, S.A.; Macarthur, D.; et al. Using high-resolution variant frequencies to empower clinical genome interpretation. *Genet. Med.* **2017**, *19*, 1151–1158.

11. Yang, X.; Song, H.; Leslie, G.; Engel, C.; Hahnen, E.; Auber, B.; Horváth, J.; Kast, K.; Niederacher, D.; Turnbull, C.; et al. Ovarian and breast cancer risks associated with pathogenic variants in RAD51C and RAD51D. *J. Natl. Cancer Inst.* **2020**, *37*, 1–9
