# Supplementary material for: Comprehensive Functional Characterization and Clinical Interpretation of 20 Splice-Site Variants of the RAD51C Gene
Source: Cancers (Basel). 2020 Dec 15;12(12):3771. doi: 10.3390/cancers12123771 (PMC7765170; doi:10.3390/cancers12123771)
Supplement: Supplementary file 1 [file cancers-12-03771-s001.zip › cancers-988386 SM proofreading/cancers-988386 Supplementary_Tables_S1_S2_S3.pdf]

Table S1. Bioinformatics analysis of *RAD51C* variants with Max Ent Score.

RAD51C VARIANTS[1]	EXON/ INTRON	MES wt	MES mut	MES score change[2]	MES *de novo* SS-mut vs. wt [3]	MES score change[2]
c.146-8A>G	ivs1/Ex2	9.5	9.4	-0.2%		
c.146-4T>C	ivs1/Ex2	9.5	9.1	-3.4%		
c.146-3C>T	ivs1/Ex2	9.5	8.7	-8.5%		
c.403T>C	Ex2	4.8	6.5	+36.3%		
c.404G>A	Ex2	4.8	-3.5	-172.7%		
c.404+6delA	Ex2/ivs2	4.8	6.4	+33.8%		
c.404+13G>A	Ex2/ivs2	4.8	4.8	0%		
c.405-6T>A	ivs2/Ex3	7.7	2.2	-72.2%	8.6 vs. 0.2	+4650% 4-nt upstream
c.571+4A>G	Ex3/ivs3	10.5	8.1	-22.5%	5.5 vs. 0.9	+525% 4-nt downstream
c.571+5G>A	Ex3/ivs3	10.5	5.8	-44.2%		
c.571+5G>T	Ex3/ivs3	10.5	6.6	-36.7%		
c.571+9G>C	Ex3/ivs3	10.5	10.5	0%		
c.572-8T>C	ivs3/Ex4	7.4	7.5	+0.4%		
c.572-7G>A	ivs3/Ex4	7.4	5.9	-21.3%		
c.572-1G>T	ivs3/Ex4	7.4	-1.2	-115.6%		
c.572A>C	Ex4	7.4	6.3	-15.2%		
c.573A>G	Ex4	7.4	7.3	-1.4%		
c.705G>T	Ex4	9.1	2.6	-72%		
c.705+5G>C	Ex4/ivs4	9.1	7.2	-20.8%		
c.705+8T>C	Ex4/ivs4	9.1	9.1	0%		
c.706-7T>C	ivs4/Ex5	11.1	10.4	-6.4%		
c.706-2A>C	ivs4/Ex5	11.1	3.1	-72.4%	3.3 vs. -2	+263.2% 10-nt downstream
c.706-2A>G	ivs4/Ex5	11.1	3.1	-71.7%	3.2 vs. -2	+254.9% 10-nt downstream
c.837+1G>A	Ex5/ivs5	8.6	0.4	-95.6%		
c.837+2T>C	Ex5/ivs5	8.6	0.8	-90.5%		

RAD51C VARIANTS[1]	EXON/ INTRON	MES wt	MES mut	MES score change[2]	MES *de novo* SS- mut vs. wt [3]	MES score change[2]
c.905-5C>G	ivs6/Ex7	8.2	6.6	-19.3%		
c.905-4T>C	ivs6/Ex7	8.2	8.1	-1.2%		
c.905-3C>G	ivs6/Ex7	8.2	-4.9	-159.4%		
c.905-2_905-1del	ivs6/Ex7	8.2	0.6	-93.3%		
c.905-2A>C	ivs6/Ex7	8.2	0.1	-98.3%		
c.965+5G>A	Ex7/ ivs7	8.7	3.8	-56.6%		
c.966-8T>C	ivs7/Ex8	7.3	8.5	+17%		
c.966-3C>A	ivs7/Ex8	7.3	4.4	-40%	5.4 vs. 5.7	-4.2%<br>3-nt upstream
c.966-2A>G	ivs7/Ex8	7.3	-0.7	-109.5%	7 vs. 5.7	+22.6%<br>3-nt upstream
c.966-2A>T	ivs7/Ex8	7.3	-1.1	-115.2%	7.6 vs. 5.7	+34%<br>3-nt upstream
c.967T>C	Ex8	7.25	6.5	-10.8%		
c.1026+5_1026+7del	Ex8/ivs8	2<br>NNSplice: 0.8	-<br><0.1	-		
c.1026+5G>T	Ex8/ivs8	2<br>NNSplice: 0.8	-<br><0.1	-		
c.1027-8T>A	ivs8/Ex9	8.6	7.4	-14.5%		
c.1029T>C	Ex9	8.6	9.1	+6.3%	4 vs. 3.9	+2.3<br>6-nt downstream

[1] Selected variants are shown in red.

[2] MES score changes (Δ%), wild type (wt) vs. mutant (mut).

[3] *De novo*: predicted creation of new alternative splice sites: mutant score vs. wild type score.

Table S2. RNA and protein impact according to sequence ENST00000337432.9 of *RAD51C*.

TRANSCRIPT [1]	RNA-HGVS	PROTEIN-HGVS	Diagrams of the splicing events [2]
**▼(E2q27)**	r. [404g>a,404_405ins404+1_404+27]<br>PTC-NMD	p.Cys135*	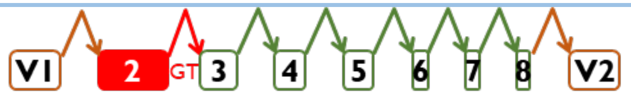
**Δ(E2q175)**	r.230_404del<br>PTC-NMD	p.Gly77Valfs*6	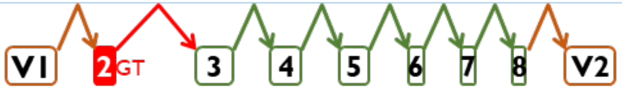
**Δ(E2q22)**	r. 383_404del<br>PTC-NMD	p.Gly128Valfs*6	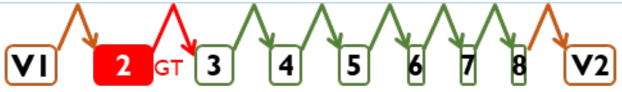
**Δ(E2)**	r. 146_404del<br>PTC-NMD	p.Glu49Valfs*6	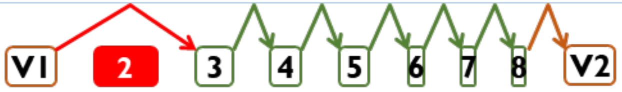
**▼(E3p4)**	r.404_405ins405-4_405-1<br>PTC-NMD	p. Cys135*	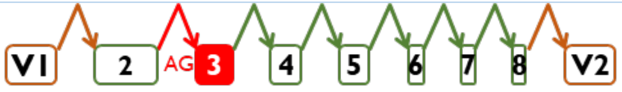
**Δ(E3)**	r. 405_571del<br>PTC-NMD	p.Cys135*	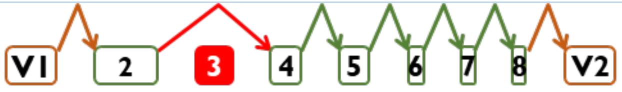
**▼(E3q4)**	r.[571+4a>g,571_572ins571+1_571+4]<br>PTC-NMD	p.Glu191Glyfs*13	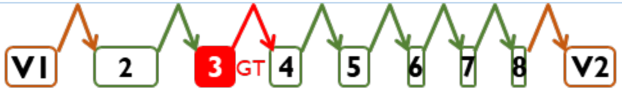
**Δ(E3q114)**	r. 458_571del	p.Gly153_Glu190del	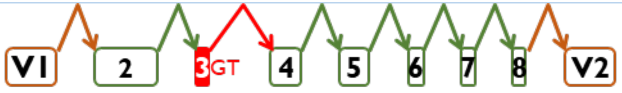
**Δ(E4)**	r. 572_705del<br>PTC-NMD	p.Glu191Glyfs*16	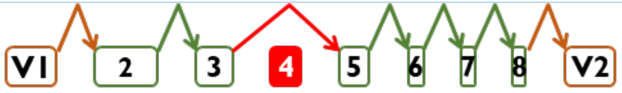
**Δ(E5p10)**	r. 706_715del<br>PTC-NMD	p.Val236*	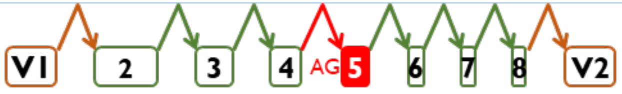
**Δ(E5p52)**	r.706_757del<br>PTC-NMD	p.Val236Metfs*10	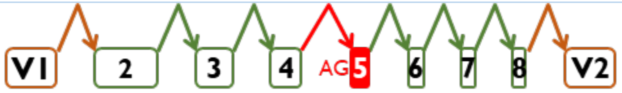

TRANSCRIPT [1]	RNA-HGVS	PROTEIN-HGVS	Diagrams of the splicing events [2]
**Δ(E5)**	r. 706_837del	p.Arg237_Val280del	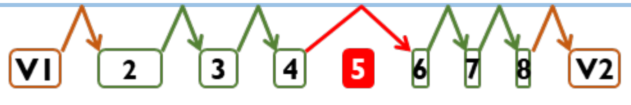
**Δ(E4_5)**	r.572_837del<br>PTC-NMD	p.Glu191Glyfs*12	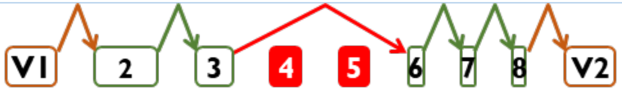
**Δ(E7)**	r.905_965del<br>PTC	p.Glu303Trpfs*41	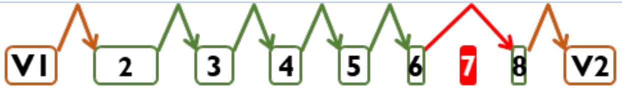
**Δ(E7_8)**	r.905_1026del<br>PTC	p.Gly302Alafs*5	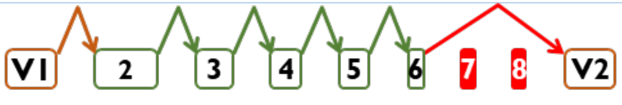
**Δ(E8)**	r.966_1026del<br>PTC	p.Arg322Serfs*22	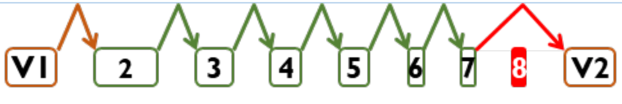
**▼(E8p3)-a**	r. [966-3c>a,965_966ins966-3_966-1]	p.Arg322dup	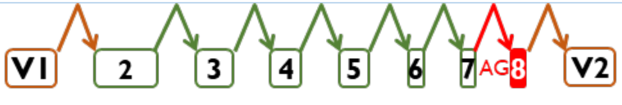
**▼(E8p3)-b**	r. [966-2a>g,965_966ins966-3_966-1]	p.Arg322delinsSerGly	
**▼(E8p3)-c**	r. [966-2a>u,965_966ins966-3_966-1]	p.Arg322delinsSerTrp	
**Δ (E8q18)**	r.1009_1026del	p.Val337_Lys342del	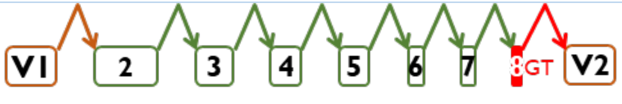
**▼(E8q41)**	r.1026_1027ins[1026+1_1026+4;1026+8_1026+44]<br>PTC	p.Pro343Valfs*4	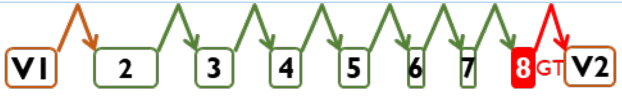
**▼(E8q44)**	r.1026_1027ins1026+1_1026+44<br>PTC	p.Pro343Valfs*5	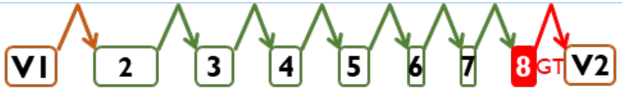

[1] Transcript annotation according to López-Perolio et al (2019) (see also in Materials and Methods).

[2] Anomalous exons and splicing events (broken lines) are indicated in red. Abnormal *de novo*/cryptic AG or GT sites are shown in red text.

Table S3. Mutagenesis primers for *RAD51C* variants

Variant	Exon/Intron	Primers (5'→3')
**c.146-3C>T**	ivs1	TTTTTTTCTTATTTTACTTTTAGAAGTTGGGATATCTAAAG
		CTTTAGATATCCCAACTTCTAAAAGTAAAATAAGAAAAAAA
**c.404G>A**	Ex2	TGTTGGAAAAACACAATTATAGTAAAATAAAGTGTTCTCCT
		AGGAGAACACTTTATTTTACTATAATTGTGTTTTTCCAACA
**c.405-6T>A**	ivs2	GTTGTTTGTCATCTTTCTGTAGACAGTATGCAGTTGGCAGT
		ACTGCCAACTGCATACTGTCTACAGAAAGATGACAAACAAC
**c.571+4A>G**	ivs3	AAACACAAGGGAGAGGGTAGGTTAGTAAATGATCTTCTT
		AAGAAGATCATTTACTAACCTACCCTCTCCCTTGTGTTT
**c.571+5G>A**	ivs3	AACACAAGGGAGAGGGTAAATTAGTAAATGATCTTCTTT
		AAAGAAGATCATTTACTAATTTACCCTCTCCCTTGTGTT
**c.572-1G>T**	ivs3	AAGAGTGTTTTGTTGTTTCATAACACCGAAAAGCTTTGGAG
		CTCCAAAGCTTTTCGGTGTTATGAAACAACAAAACACTCTT
**c.705G>T**	Ex4	TTCCTTTCAGAACACTCAAATGTATGAGTCAGACTACTGAA
		TTCAGTAGTCTGACTCATACATTTGAGTGTTCTGAAAGGAA
**c.705+5G>C**	ivs4	TTCAGAACACTCAAAGGTATCAGTCAGACTACTGAAATGTA
		TACATTTCAGTAGTCTGACTGATACCTTTGAGTGTTCTGAA
**c.706-2A>C**	ivs4	ATATTATCTCTTCTGTATTTCGGTTCGACTAGTGATAGTGG
		CCACTATCACTAGTCGAACCGAAATACAGAAGAGATAATAT
**c.706-2A>G**	ivs4	ATATTATCTCTTCTGTATTTGGGTTCGACTAGTGATAGTGG
		CCACTATCACTAGTCGAACCCAAATACAGAAGAGATAATAT
**c.837+2T>C**	ivs5	AAATAATCACAGATTAGCTGCAAGTATTAACTAGTGAAGAG
		CTCTTCACTAGTTAATACTTGCAGCTAATCTGTGATTATTT
**c.905-3C>G**	ivs6	GTTATTATGTTTTTTACTCTGAGGGGAAAGTTGGGGACATG
		CATGTCCCCAACTTTCCCCTCAGAGTAAAAAACATAATAAC
**c.905-2A>C**	ivs6	TTATTATGTTTTTTACTCTCCGGGGAAAGTTGGGGACATGC
		GCATGTCCCCAACTTTCCCCGGAGAGTAAAAAACATAATAA
**c.905-2_905-1del**	ivs6	TACAGTTATTATGTTTTTTACTCTCGGGAAAGTTGGGGACATGCTG
		CAGCATGTCCCCAACTTTCCCGAGAGTAAAAAACATAATAACTGTA
**c.965+5G>A**	ivs7	GGGACCGAAAGCAAAGGTCAATACAGAAACAAGTTAATAAC
		GTTATTAACTTGTTTCTGTATTGACCTTTGCTTTCGGTCCC
**c.966-3C>A**	ivs7	ATTTATTCTTTTTCTTTAAGAAGGTTGGCAACATTGTACAA
		TTGTACAATGTTGCCAACCTTCTTAAAGAAAAAGAATAAAT
**c.966-2A>G**	ivs7	TTTATTCTTTTTCTTTAAGCGGGTTGGCAACATTGTACAAG
		CTTGTACAATGTTGCCAACCCGCTTAAAGAAAAAGAATAAA

**c.966-2A>T**	ivs7	TTTATTCTTTTTCTTTAAGCTGGTTGGCAACATTGTACAAG
		CTTGTACAATGTTGCCAACCAGCTTAAAGAAAAAGAATAAA
**c.1026+5_1026+7del**	ivs8	GAATGCACAGTACTGTTTCAAATCAAAGTCATTATTTGATTAGAGTGGGATTTTGATATT
		AATATCAAAATCCCACTCTAATCAAATAATGACTTTGATTTGAAACAGTACTGTGCATTC
**c.1026+5G>T**	ivs8	ACTGTTTCAAATCAAAGTCATTATTATTTGATTAGAGTGGG
		CCCACTCTAATCAAATAATAATGACTTTGATTTGAAACAGT
